# Glocal Clinical Registries: Pacemaker Registry Design and Implementation for Global and Local Integration – Methodology and Case Study

**DOI:** 10.1371/journal.pone.0071090

**Published:** 2013-07-25

**Authors:** Kátia Regina da Silva, Roberto Costa, Elizabeth Sartori Crevelari, Marianna Sobral Lacerda, Caio Marcos de Moraes Albertini, Martino Martinelli Filho, José Eduardo Santana, João Ricardo Nickenig Vissoci, Ricardo Pietrobon, Jacson V. Barros

**Affiliations:** 1 Heart Institute (InCor) – Clinics Hospital of the University of São Paulo Medical School, São Paulo, Brazil; 2 Department of Surgery, Duke University Medical Center, Durham, North Carolina, United States of America; 3 Department of Cardiovascular Surgery, Heart Institute (InCor) – Clinics Hospital of the University of São Paulo Medical School, São Paulo, Brazil; 4 Department of Cardiology, Heart Institute (InCor) – Clinics Hospital of the University of Sao Paulo Medical School, São Paulo, Brazil; 5 Computing Institute of the Federal University of Alagoas, Alagoas, Brazil; 6 Research Fellow – Department of Anesthesiology, Duke University Medical Center, Durham, North Carolina, United States of America; 7 Pontific Catholic University of Sao Paulo, Sao Paulo, Brazil; 8 Medicine Department, Faculdade Ingá, Maringá, Brazil; 9 Department of Surgery, Duke University Medical Center, Durham, North Carolina, United States of America; 10 Clinics Hospital of the University of São Paulo Medical School, São Paulo, Brazil; University Medical Center Utrecht, The Netherlands

## Abstract

**Background:**

The ability to apply standard and interoperable solutions for implementing and managing medical registries as well as aggregate, reproduce, and access data sets from legacy formats and platforms to advanced standard formats and operating systems are crucial for both clinical healthcare and biomedical research settings.

**Purpose:**

Our study describes a reproducible, highly scalable, standard framework for a device registry implementation addressing both local data quality components and global linking problems.

**Methods and Results:**

We developed a device registry framework involving the following steps: (1) Data standards definition and representation of the research workflow, (2) Development of electronic case report forms using REDCap (Research Electronic Data Capture), (3) Data collection according to the clinical research workflow and, (4) Data augmentation by enriching the registry database with local electronic health records, governmental database and linked open data collections, (5) Data quality control and (6) Data dissemination through the registry Web site. Our registry adopted all applicable standardized data elements proposed by American College Cardiology / American Heart Association Clinical Data Standards, as well as variables derived from cardiac devices randomized trials and Clinical Data Interchange Standards Consortium. Local interoperability was performed between REDCap and data derived from Electronic Health Record system. The original data set was also augmented by incorporating the reimbursed values paid by the Brazilian government during a hospitalization for pacemaker implantation. By linking our registry to the open data collection repository Linked Clinical Trials (LinkedCT) we found 130 clinical trials which are potentially correlated with our pacemaker registry.

**Conclusion:**

This study demonstrates how standard and reproducible solutions can be applied in the implementation of medical registries to constitute a re-usable framework. Such approach has the potential to facilitate data integration between healthcare and research settings, also being a useful framework to be used in other biomedical registries.

## Introduction

Over the past few years, the worldwide volume of healthcare and clinical research data generated has been significantly expanded [1]–[3]. Data sources now encompass multiple registries and clinical trials as well as the progressive implementation of hospital administration and electronic health record (EHR) systems [1]–[6]. As a special case of data collection systems, medical device registries have been essential to guide improvements in technology and to facilitate the refinement of patient selection in order to maximize outcomes with current and new device options [4], [5], [7], [8]. Studies derived from well-designed and well-conducted medical devices registries can provide a real-world view of clinical practice, patient outcomes, safety, comparative effectiveness and cost effectiveness and may strengthen a number of evidence development and decision making process [4], [5], [7]–[14].

Despite its huge potential for both biomedical research as well as the potential to positively affect clinical practice and healthcare policies, medical registries are frequently surrounded by process problems that substantially decrease their value [4], [15], [16]. These include missing data and poor data quality, which is related to how the research component of the registry is connected to clinical workflow and how personnel involved in the data collection are trained [4]. Compatibility problems with other health registries or publicly available data sets, which are associated with how data elements are structured and defined to accomplish the registry's intended purposes are other weakness presented in large quantity of electronic medical registries [4], [17]–[20].

Although web-based electronic data capture (EDC) systems have become more prevalent across the globe, the data collection for research purposes is still a challenging process [4], [20]–[22]. Lack of harmonization between the clinical and research workflows is time consuming for both clinical staff and patients [23], [24]. In addition, many hospitals and healthcare facilities that participate in studies present different data capture systems for both healthcare and research settings resulting in effort duplication, ultimately leading to data inconsistency [4], [18]–[20].

Adopting standardized data elements and a common terminology is arguably the key to facilitate the exchange of data across studies and to promote interoperability between different EHRs systems [4], [17]–[20]. The objective of this study is therefore to describe a reproducible, highly scalable, standard framework for a device registry implementation addressing both local data quality components as well as global linking problems. In the first section of our article we set the theoretical background, while in the second section we provide a clinical use case involving a pacemaker registry implementation designed to systematically collect interpretable long-term safety and outcomes data.

## Methods

### Registry description

The Pacemaker Registry Open Data Collection is derived from the SAFE-LV PACE randomized trial (“Safety and the Effects of Isolated Left Ventricular Pacing in Patients With Bradyarrhythmias,” ClinicalTrials.gov study ID NCT01717469). This randomized controlled study is being conducted to compare the effects of conventional right ventricular (RV) pacing versus left ventricular (LV) pacing in patients with atrioventricular block. Our main hypothesis is that isolated LV pacing through the coronary sinus can be used safely and provide greater hemodynamic benefits to patients with atrioventricular block and normal ventricular function who require only the correction of heart rate. Specifically, our aims are to evaluate the safety, efficacy and the effects of LV pacing using active-fixation coronary sinus lead – *Attain StarFix® Model 4195 OTW Lead*, compared to RV pacing in patients with implantation criteria for conventional pacemaker stimulation.

In this registry we are creating a large and interoperable database to report pacemaker long-term outcomes. All clinical data stored will maintain full patient confidentiality according to Good Clinical Practices (GCP) and the Health Insurance Portability and Accountability Act (HIPAA) [25] and will be freely available to allow collaboration between researchers around the world. Main advantages of this open data collection include the incentive for interdisciplinary and multi-institutional collaborations, along with the creation of clinical and policy measures in a more timely manner.

### Glocal registry methodology

The Institutional Review Board of the Clinics Hospital of the University of São Paulo Medical School (São Paulo, Brazil) approved this study. All participating subjects provided written informed consent. All elements in this article comply with a reproducible research protocol [26].

**Figure 1:**
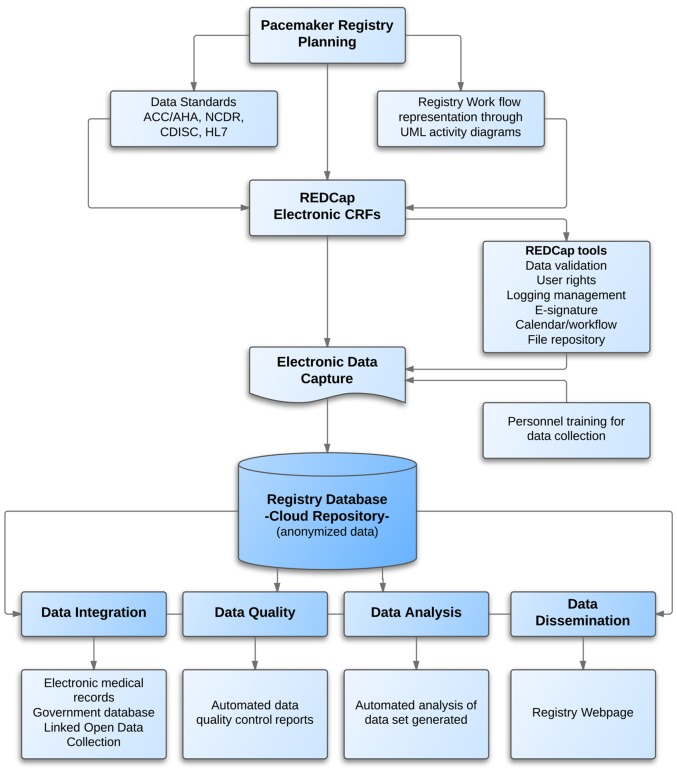
Registry processes representation. Legend: ACC/AHA =  American College of Cardiology/ American Heart Association; CDISC =  Clinical Data Interchange Standards Consortium; CRF =  Case Report Form; HL7 =  Health Level Seven; NCDR =  National Cardiovascular Data Registry; REDCap =  Research Electronic Data Capture.

The device registry implementation comprised a group of generic processes successfully applied to project management, including the initiation, planning, execution, monitoring and controlling, and closing. The sequence included: (1) data planning used to define the common data standards and terminology as well as the representation of the research workflow, (2) development of electronic case report forms using REDCap (Research Electronic Data Capture), (3) the process of data collection according to the clinical research workflow, (4) the aggregation between the registry data and other systems, (5) data quality control and data analysis using statistical methods and, finally (6) the data dissemination through the registry Web site (Figure 1).

### Defining Data Elements

Over the last few years, the American College of Cardiology (ACC) and the American Heart Association (AHA) have started an initiative to develop and publish clinical data standards that can be used in a variety of data collection efforts for a range of cardiovascular conditions [27], [28]. The ACC/AHA Writing Committee to Develop Clinical Data Standards for Electrophysiology was charged with providing standard definitions to relevant terms in the care of patients with a diagnosis of arrhythmia and implanted cardiac electronic devices [29].

Our registry adopted all applicable data elements and definitions in accordance with ACC/AHA available published data standards, including those developed for Electrophysiology, Atrial Fibrillation, Acute Coronary Syndromes, Heart Failure, and Cardiac Imaging [29]–[33]. Other data sources included data elements from large device clinical trials and registries, such as CTOPP (Canadian Trial of Physiologic Pacing) [34], MOST (Mode Selection Trial in Sinus Node Dysfunction) [35], COMPANION (Comparison of Medical Therapy, Pacing, and Defibrillation in Heart Failure) [36], REVERSE (REsynchronization reVErses Remodeling in Systolic Left vEntricular Dysfunction) [37]. We also reviewed case report forms, data elements, and definitions from international data collection efforts. Examples of these data sources include the ACC National Cardiovascular Data Registry (NCDR) [38], [39], Health Level Seven International (HL7) [40], Clinical Data Interchange Standards Consortium (CDISC) [41] and Cancer Data Standards Registry and Repository (caDSR) [42], [43]. Finally, we also included standardized definitions for clinical endpoints and adverse events in cardiovascular trials from the US Food and Drug Administration (FDA) [44].

### Defining the Registry Workflow – Clinical Activity Model

Based on discussions with practicing clinicians and participatory observation of the clinic by two of the authors (KRS and RC), UML (Unified Modeling Language) activity diagram models were prepared to represent the clinical registry as well as the data collection workflow. A comparison of these clinical and data collection workflow models was then conducted to ensure the detection of potential areas where the activities related to data collection might not be in perfect alignment with the activities executed in the daily clinical workflow, ultimately leading to data quality issues, rework, and other processes inefficiencies. These diagrams were modeled according to UML version 2.0 [45]. All activity diagrams were created using ArgoUML (version 0.34) [46].

### Electronic Data Collection

**Figure 2:**
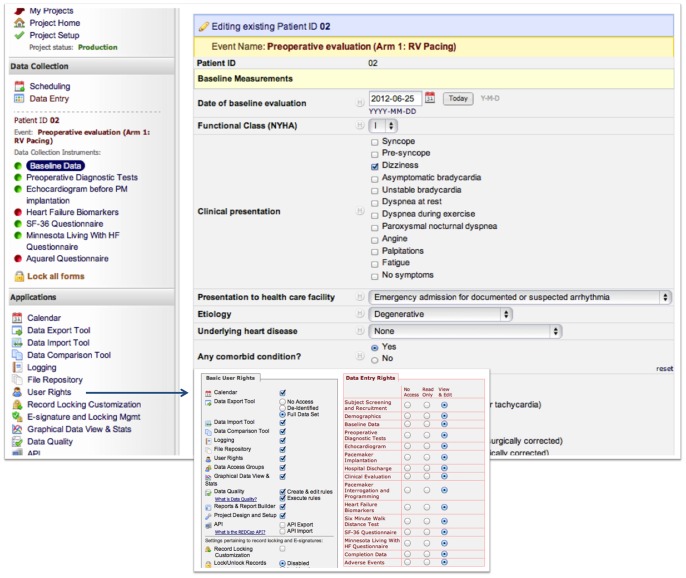
REDCap Data Entry. Footnote: Case report forms are accessible to users who have sufficient access rights and it contains field-specific validation code sufficient to ensure data integrity.

Once the absence of potential workflow dissonance was ensured through the modeling, electronic case report forms (CRFs) were developed using the REDCap [21] EDC tool hosted at a local server within the firewall of the University of São Paulo Health System. REDCap is a secure web-based software and workflow methodology for electronic collection and management of research data (Figure 2). Among other characteristics it provides (1) an intuitive interface for validated data entry, with automated data type and range checks; (2) audit trails for tracking data manipulation and export procedures, (3) automated data export procedures to common statistical packages, and (4) procedures for importing data from external sources [21]. Research coordinators performed data capture by using a tablet computer through a secure Wi-Fi, ultimately allowing for portable data collection at the point of care.

### Personnel Training for Data Collection

We performed a semi-structured training with the clinical research coordinators. Our goal was to provide a general overview of the registry database, while concurrently identifying specific factors which could compromise the integrity of the data collection. To ensure a standardized and consistent data collection we developed a standard operating procedure (SOP) specifically related to the primary data collectors tasks. This SOP provides a description of all data elements collected as well as the sources used to obtain the data.

After the training process, the data entry activities of clinical research coordinators were closely monitored for three months by the principal investigators (RC and KRS) to assess whether data collection was conducted according to the study protocol. We used the REDCap report tool for monitoring and querying patient records. Corrective actions were taken to address problems related to data inconsistency and missing information, involving retraining and immediate feedback on issues such as missing, out-of-range values and logical inconsistencies.

### Data Augmentation

The purpose of data augmentation was to augment variables to the research component of our pacemaker registry data sets from clinical and administrative sources, ultimately enhancing our ability to evaluate important research questions. The original dataset was augmented by incorporating data derived from three different instances: (1) EHR from the Clinics Hospital of the University of São Paulo Medical School (HCFMUSP); (2) Brazilian governmental database and (3) Linked Open Data (LOD) Collection. In the following section we describe the methodology used to perform the data integration across these sources.

#### Linking Registry Data with Local Electronic Health Records

Whereas the study is being conducted at Heart Institute (InCor) – Clinics Hospital of the University of São Paulo Medical School, all demographics characteristics as well as healthcare information are available in several databases from legacy systems. Given the heterogeneity of these multidatabase systems, each patient has a unique identifier (ID) making it possible to associate the right health information with the right individual.

In order to avoid duplicate data entry, the EHR from the Clinics Hospital of the University of São Paulo Medical School (HCFMUSP) was integrated to the EDC through the REDCap API (Application Program Interface). The REDCap API is an interface that allows external applications to connect to REDCap remotely, and it is used for programmatically retrieving or modifying data or settings within REDCap. As the API is a built-in feature of REDCap, no installation is required and this tool implements the use of tokens as a means of authenticating and validating all API requests that are received. In addition, the API also implements data validation when the API is used for data import purposes in order to ensure that only valid data will be stored. By using the REDCap API, it was possible to retrieve useful demographic information directly from the sources of hospital systems.

#### Linking Registry Data and Governmental Database

The original data set was augmented by incorporating publicly available data from the Brazilian governmental database known as DATASUS (Information Technology Department of the Brazilian Unified Health System, or SUS) [47]. This database produces a significant volume of information and provides the reimbursed values by the government for public healthcare organizations in both inpatient and outpatient care systems. For inpatients the common unit describing hospital charges is the hospital admission authorization, which is in accordance with the Hospital Information System. In addition, this database provides other information such as: reasons for hospitalization, length of hospital stay, socio-demographic characteristics, diagnoses, medical procedures, healthcare service providers and also the values paid for each procedure performed by public healthcare organizations.

We created a repository to store all anonymized data derived from DATASUS under the Amazon Elastic Compute Cloud – Amazon EC2 [48]. This repository hosts a MySQL server where the database is available in a normalized format. In this repository, we have stored a set of databases that comprises the basis for hospital accountability of the Brazilian Unified Health System (SUS), in which all diagnoses and procedures are coded according to ICD-10. Through this repository was possible to retrieve useful information such as reimbursed values by the government for pacemaker implantation as well as length of hospital stay.

This database is available in CSV (comma separated values) format files and all data are updated monthly on the Web site of DATASUS.

#### Linking Registry Data with Linked Open Data Collections

In addition, we also enriched our registry by adding open semantic web data source for clinical trials named Linked Clinical Trials (LinkedCT) [49]. Each clinical trial in this database is associated with a brief description of the trial, related conditions, interventions, eligibility criteria, sponsors, locations and other additional information. This mapping was implemented by means of a SPARQL query interconnecting our dataset with the Linked Life Data (LLD) endpoint [50]. This approach enables the identification of correlated clinical trials and investigators in order to generate new opportunities for scientific collaboration.

#### Data de-identification

All data – including images, lab tests and any associated information – were de-identified before insertion into the repository as required by HIPAA (Health Insurance Portability and Accountability Act) regulations to ensure that protected health information (PHI) was not inappropriately used or disclosed [25]. The de-identification was performed by indicating a variable as PHI element during the project development process in REDCap and also by selecting those variables prior to exporting the data.

#### Data modeling resources

Our data repository also contains an instance of the R statistical language (version 2.15.1) [51], along with the RStudio Server version 0.96 IDE (integrated development environment). Through this infrastructure users can easily manipulate statistical scripts, generate reports, and directly upload them to the server on the same environment.

### Data quality control, association and prediction reports

We established a system to generate automated data quality control and prediction reports based on the R statistical language. This system involves a set of packages enabling literate programming and reproducible research standards to automatically transform the statistical results into a real-time reports deployed in HTML (HyperText Markup Language) and PDF (Portable Document Format), both available from our central Web site [52]. Reports are created using the knitr package [53] and the Markdown language [54] in combination with R [51]. Specifically, we use R Markdown files with subsequent transformations to HTML and PDF performed through pandoc [55]. Documents are then presented on our Web server through the rApache package [56], ultimately ensuring that data quality reports are maintained up to date. Scripts for all of our procedures are available at our Github repository [57].

Association reports are also provided as a mechanism for exploratory graphical analysis. Among them, we included the MINE (Maximal Information-based Nonparametric Exploration) algorithm [58], a sophisticated, robust algorithm used for exploratory analyses. Extensive use of exploratory graphical methods is facilitated by the use of the R package ggplot2 [59], along with other methods for data manipulation. Additional integrated services included the use of BigQuery [60] for manipulating large data sets as well as Google prediction services [61].

### Open Design

In order to provide incentives for other researchers to join the collaboration and start creating analyses using the dataset, we have created a special section on our Web site [52] and Github repository [57] with a data dictionary and de-identified data sets in an Open Data format.

## Results

### Pacemaker Registry Detailed Use Case

**Figure 3:**
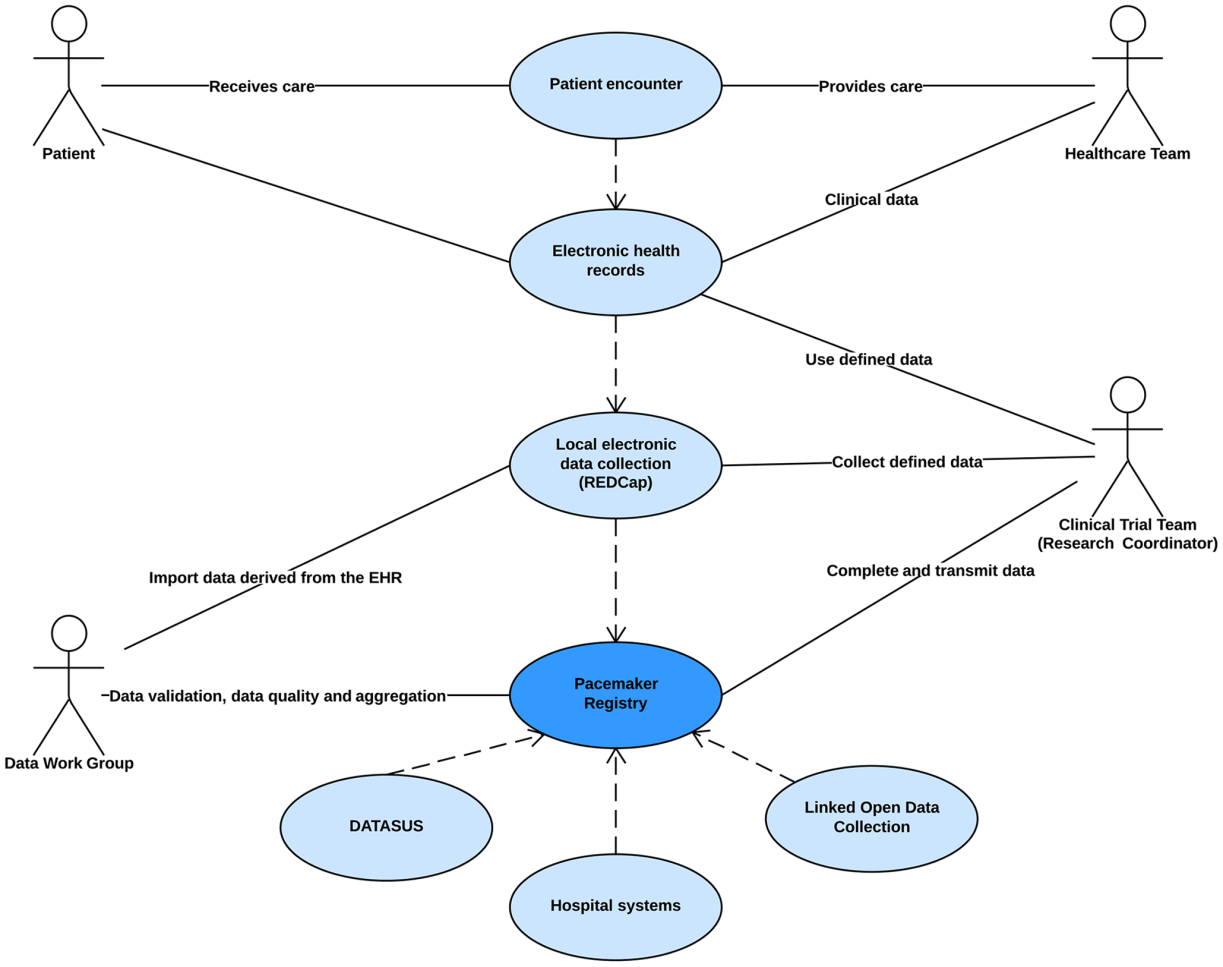
Pacemaker Registry Use Case Stakeholders.

The use case model describes the process of information exchange involved in our pacemaker registry, detailing the infrastructure developed to enable interoperability between the EHR and REDCap. For this use case, the workgroup has prioritized the electronic data capture of standardized data elements in order to leverage a core set of widely useful clinical data from EHR systems to increase the effectiveness and efficiency of clinical research activities. The following diagram (Figure 3) illustrates the stakeholders involved in the processes described in this use case.

**Figure 4:**
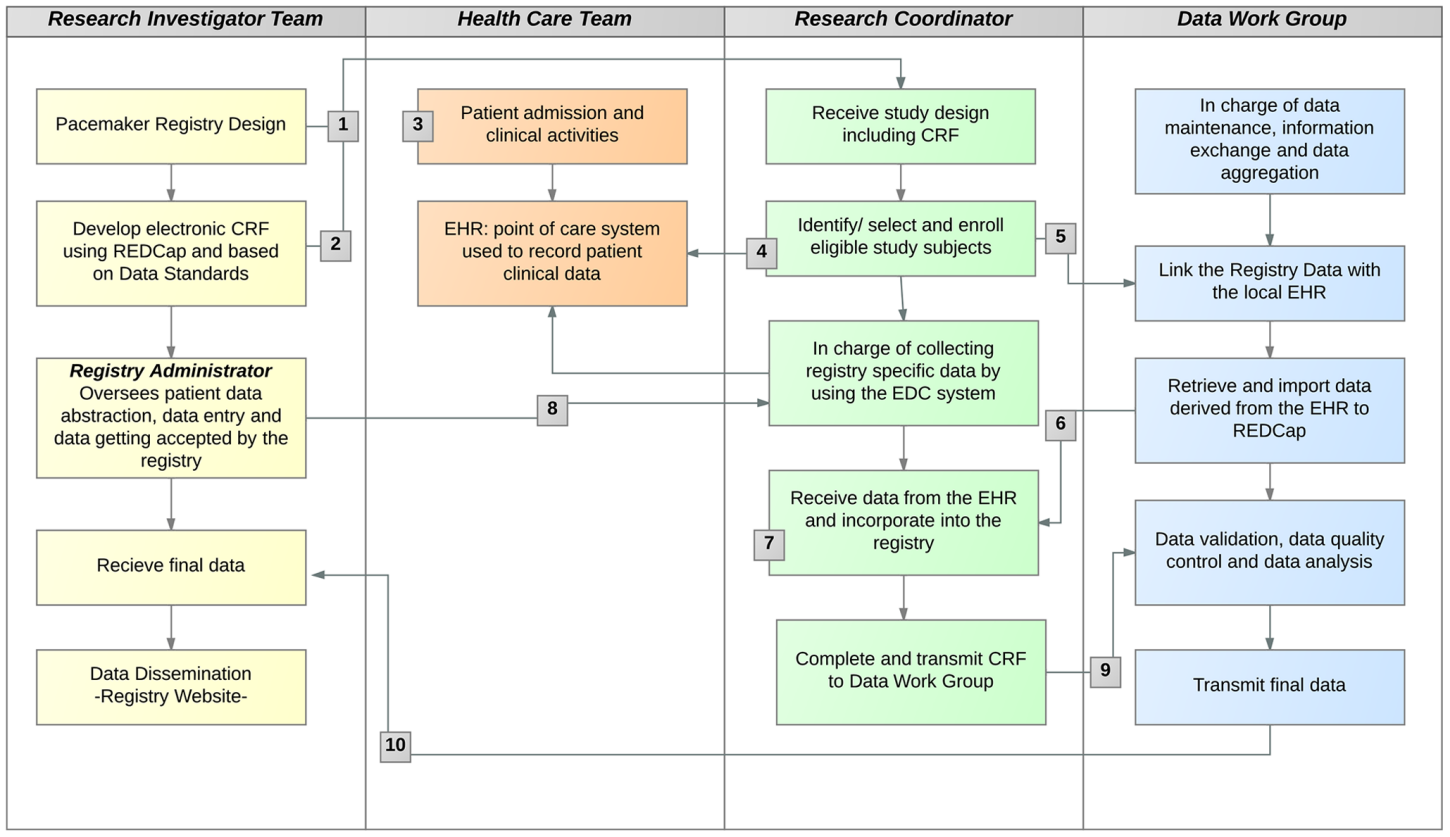
Pacemaker Registry Activity Diagram to Support Data Exchange between EHR system and REDCap. Footnotes: (1) The study design is communicated to Clinical Trial Team, specifically to the Research Coordinator. (2) Case Report Form (CRF) is developed using the REDCap EDC tool hosted at a local server within the firewall of the University of São Paulo. Once built the CRF, the Registry Administrator will assign users rights for system access. (3) Patient is admitted to facility and the healthcare team entered demographic and clinical data into the EHR. (4) Research coordinators identify eligible study subjects by consulting the EHR patients records. (5) After patient enrollment, a REDCap API request is send to the Data Work Group for retrieving and importing socio-demographic information directly from the sources of hospital systems. (6), (7) Information is exchanged between the EHR and REDCap. (8) Registry administrator oversees all data collected by research coordinators. (9) CRF is transmitted from the research coordinators to the Data Work Group for data validation, data quality control e data analysis. (10) Data Work Group transmits CRF and aggregated data to the Research Team and Registry Administrator.

Indication of pacemaker implantation in a patient presenting bradyarrhythmia is the condition determining the start of this use case. By assessing patients, healthcare team entered demographic and clinical data into the EHR. Research coordinators identify subjects for the study based upon whether they meet the protocol eligibility criteria. Once study subjects were enrolled in the study, a core set of data may be exchanged from the clinical EHR system to REDCap as previously described in the “*Linking Registry Data with Local Electronic Health Records”* section. Research coordinators were responsible for completing the data retrieve from the EHR into the REDCap as well as for electronic data capture of additional study-specific data during the course of the study. All collected data is transmitted to the Data Work Group for validation and later to the Research Investigators Team. The Data Work Group is responsible for data maintenance, information exchange, and data aggregation with other databases. (Table 1, Figure 4).

**Table 1:** Pacemaker Registry Use Case Description.

Use Case	Contextual Description
Description	This use case describes the steps involved in the data collection and data aggregation in order to develop a comprehensive pacemaker registry
Participants	Patients, Physicians, Nursing staff, Clinical Trial Team, Data Work Group, , Registry Administrator, Research Investigators Team
Trigger	Patient presenting bradyarrhythmia and indication to pacemaker implantation according to current guidelines is admitted to facility
Precondition	Patient is enrolled in the study. Research coordinators start the data collection process.
Post conditions	Data work group receives the data and perform the validation, aggregation and storage.
Normal Flow	Patient demographic, history and clinical information are collected. Patient is submitted to preoperative evaluation and all findings are recorded in the electronic health record.
	Clinical Trial Team collects registry specific data as identified in the registry protocol using REDCap.
	Data work group performs data exchange between EHR and REDCap, data validation, data quality control, data analysis, data aggregation and storage in the cloud repository.

EHR =  Electronic health record; REDCap =  Research Electronic Data Capture.

### Pacemaker Registry Workflow

**Figure 5:**
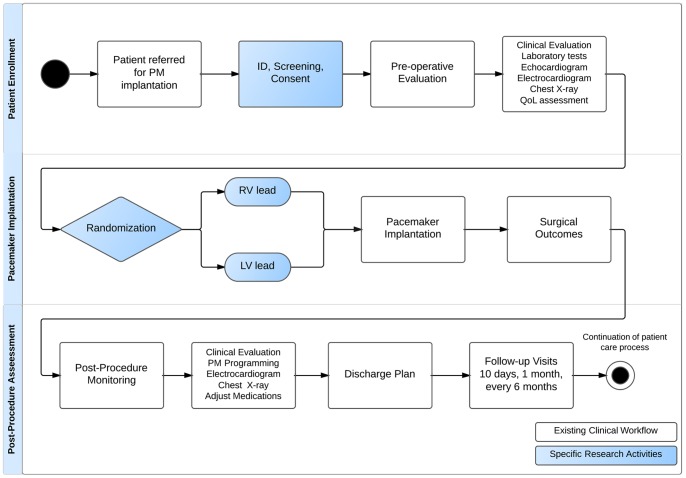
Pacemaker Registry Activity Diagram. Footnote: This figure represents the alignment between clinical (white flowchart) and research (blue flowchart) workflows.

The registry UML-AD represents the activity workflow associated with data capture for subjects meeting study criteria for inclusion in our registry. This workflow illustrates the process of patient care throughout diagnosis, assessment, treatment, and long-term monitoring of patients undergoing pacemaker implantation. In addition, this registry workflow was aligned with the clinical workflow to enhance quality of the data captured and also facilitate understanding of the clinical care and research processes as a common reference by both clinicians and technologists (Figure 5).

### Clinical Data Standards

Most variables contained in the CRFs were based on standardized data elements proposed by ACC/AHA Clinical Data Standards [28]–[33]. We also used variables derived from cardiac devices randomized trials [34]–[37], as well as NCI Thesaurus and CDISC data standards [42], [43]. (Table 2) The authors added specific pacemaker data elements which are not yet available in the standardization sources used in this study. Data standards for each variable class are detailed in Supporting Information (Table S1).

**Table 2:** Pacemaker Registry Clinical Data Standards Elements.

Variable Class	Source of Standardization
Patient Identifiers	NCI Thesaurus [42], [43]
Patient Demographics	NCI Thesaurus, ACC/AHA [28]–[33]
Patient History	ACC/AHA [28], [29]
Laboratory Tests	ACC/AHA [28]–[33]
Specific Electrocardiogram Patterns	ACC/AHA [29], [30], Trials [34]–[37]
Chest Radiography	ACC/AHA [32], data elements under development
Echocardiography before and after Pacemaker Implantation	ACC/AHA [29], [30], [33], Prospect trial [62]
Pacemaker Implantation	ACC/AHA [29], Trials [34]–[37], data elements under development
Hospital Discharge	ACC/AHA [28]–[33], Trials [34]–[37], data elements under development
Follow-up Evaluations	ACC/AHA [29], Trials [12], [13], [34]–[37], data elements under development
Pacemaker Interrogation and Programming	Data elements under development
Heart Failure Biomarkers	Data elements under development
Six Minute Walk Distance Test	ACC/AHA [29], ATS [63], data elements under development
SF-36 Questionnaire	ACC/AHA [32], SF-36 [64]
Minnesota Living With Heart Failure Questionnaire	ACC/AHA [32], MLWHF [65]
Aquarel Questionnaire	Aquarel [66]
Completion Data	CDISC [41], ACC/AHA [28]–[33], data elements under development
Adverse Events	ACC/AHA [28]–[33], CDISC [41]

ACC/AHA =  American College of Cardiology/American Heart Association; ATS =  American Thoracic Society; CDISC =  Clinical Data Interchange Standards Consortium; NCI =  National Cancer Institute; SF-36 =  Short-form 36 questionnaire.

### Data quality control and prediction reports

Analysis of the data quality was performed in three instances: (1) Exploratory analysis of missing data to map the frequency, location and effect of missing data in a given dataset or variable class; (2) Descriptive statistics (mean, standard deviation and frequency) of subsets in different moments of data collection to establish a confidence limit; (3) Benford's Law or first-digit law in order to monitor for possible data fabrication. Data association and prediction plots were generated based on boxplots for reports of numeric data and association plots for categorical data. We also used the application of the MINE algorithm [58] to explore the association between two pairs of numeric variables, both linear and nonlinear. Corresponding code for the generation of automated reports in HTML and PDF is available in our Github repository [57] and graphs for each data analysis performed are available under Supporting Information (Report S1).

### Data Augmentation

Scripts for the data augmentation are available under our Github repository [57]. A full report in HTML and PDF formats converted from our script is available on our central web site. As an example of an augmented variable, a summary of reimbursed values paid by the government during a hospitalization for pacemaker implantation and the length of hospital stay are presented in Table 3. The data in this table indicate the variation in costs and length of hospital stay according to the geographic region. Additional details about each Brazilian state are provided under Supporting Information (Table S2).

**Table 3:** Reimbursed values paid by Brazilian government for pacemaker implantation according to geographic region.

Geographic Region	Reimbursed values per day, USD* (mean)	Total reimbursed values, USD* (mean)	Length of hospital stay
Centre west	$2,225.30	$4,000.56	3.2
North	$1,156.99	$3,685.86	7.0
Northeast	$2,395.56	$3,966.76	2.9
South	$1,938.42	$3,988.23	4.2
Southeast	$2,144.09	$4,005.59	3.7

* Brazilian real (BRL) converted to US Dollar (USD) in December 2, 2012.

1 BRL  =  0.468061 USD.

1 USD  =  2.13647 BRL.

Table 4 shows a total of 130 clinical trials available at LinkedCT which are potentially associated with this pacemaker registry. The SPARQL endpoint is provided into our Github repository [57], as well as a full report with detailed conditions, interventions, eligibility criteria, sponsors, locations and other additional information. Additional details about each clinical trial are provided under Supporting Information (Table S3).

**Table 4:** Cardiac Pacemaker Clinical Trials available at LinkedCT.

Status	Intervention	Condition	Count
Completed	Pacemaker	Atrial fibrillation	4
Terminated	Pacemaker	Atrial fibrillation	1
Recruiting	Pacemaker	Heart Block	4
Active, not recruiting	Pacemaker	Heart Block	2
Not yet recruiting	Pacemaker	Heart Block	1
Enrolling by invitation	Pacemaker	Heart Block	1
Recruiting	Pacemaker	Bradyarrhythmia	15
Active, not recruiting	Pacemaker	Bradyarrhythmia	8
Not yet recruiting	Pacemaker	Bradyarrhythmia	4
Enrolling by invitation	Pacemaker	Bradyarrhythmia	1
Completed	Pacemaker	Bradyarrhythmia	19
Terminated	Pacemaker	Bradyarrhythmia	2
Recruiting	Pacemaker or CRT	Heart failure	22
Active, not recruiting	Pacemaker or CRT	Heart failure	10
Not yet recruiting	Pacemaker or CRT	Heart failure	4
Enrolling by invitation	Pacemaker or CRT	Heart failure	1
Completed	Pacemaker or CRT	Heart failure	25
Terminated	Pacemaker or CRT	Heart failure	5
Suspended	Pacemaker or CRT	Heart failure	1

CRT =  cardiac resynchronization therapy.

### Open Design and Data Dissemination

**Figure 6:**
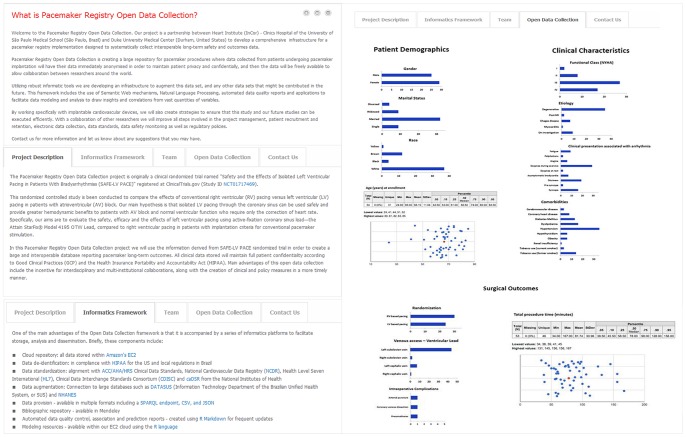
Pacemaker Registry Website. Figure 6A – Pacemaker Registry Website – General Information. Figure 6B – Pacemaker Registry Website – Open Data Collection.

The Open Data collection includes de-identified raw data sufficiently enough to describe the demographic and clinical profile of patients submitted to pacemaker implantation as well as surgical and clinical outcomes associated with both study interventions (Table 5). The following illustration (Figure 6) is derived from our Web site, in which all data will be updated every six months.

**Table 5:** Pacemaker Registry Open Data Collection.

Variable Class	Research Questions	Data available
Patient Demographics	Demographic patient profile	Age, gender, race, ethnicity, geographic localization, insurance payer, presentation to healthcare facility
Clinical Characteristics	Clinical patient profile	Presentations associated with arrhythmia, arrhythmia history, specific ECG patterns, NYHA functional classification, etiology, underlying heart disease, comorbid conditions, history of cardiovascular disease, history of non-cardiovascular diseases
Surgical outcomes	Success rate of ventricular lead implantation and surgical complications in both study groups	Surgical procedure performed, total ventricular lead positioning time, total procedure time duration, procedure success rate, intraoperative complications
Clinical outcomes	Heart failure and ventricular dysfunction incidence in both study groups	Clinical manifestations after PM implantation, functional class (NYHA) after PM implantation, cardiovascular symptoms after PM implantation, left ventricular ejection fraction after PM implantation

ECG =  electrocardiogram; NYHA =  New York Heart Association; PM =  pacemaker.

## Discussion

The foundational work to create this pacemaker registry is part of a broader program to address the lack of data interoperability between the clinical and research settings. In this manuscript, we describe the infrastructure behind our Pacemaker Registry involving a diversity of steps such as: a comprehensive database planning, the alignment between research and clinical workflows, the adoption of clinical data standards, the development of electronic case report forms using REDCap, the aggregation between registry data and other systems and, finally the open data collection dissemination by the registry Web site.

This methodological study is also an effort to implement glocal (global and local) data integration through a reproducible research protocol, which can be applied to other medical registries. In the scope of our study, “global” integration involves the adoption of global data standards and data interchange to facilitate information sharing within and across institutions. “Local” integration implies in integrating workflow between research and healthcare settings, and also in the interoperability between EHR and EDC systems.

Successful registries depend on a sustainable workflow model that should be aligned to the daily clinical practice with minimal disruption [1]–[8]. Previous studies suggested [23], [24], [44] that workflow efficiency is a valuable factor for enhancing data quality and integrity since inefficient process may result in errors related to data collection and transcription, as well as unnecessary redundancy in the data collection [4], [5], [18]–[20]. In our study, we have made an effort to align the EDC system with the clinical workflow and we are currently working on the integration between EHR and EDC systems. In particular, the REDCap functionalities allowed us to develop an efficient interface between healthcare and research data collection, enabling the reuse of EHR data.

For the development of interoperability and internationalization of our registry we focused firstly on data standards by using all existing standards terminologies whenever possible. It included all standard terminologies published by ACC/AHA [28]–[33], as well as data elements derived from large device clinical trials and other sources as NCDR [38], [39], HL7 [40], CDISC [41] and caDSR [42], [43]. The use of established data standards is crucial for semantic interoperability between information systems, which will be increasingly important as the use of electronic health information system is becoming widely available around the globe. It is also important to consider that the adoption of data standard terminologies not only improves the efficiency in establishing registries but also promote more effective sharing, combining, or linking of data sets from different sources and institutions. In addition, the use of well-defined standards for data elements ensures that the meaning of data captured in different systems is the same.

Several different methods can be applied for the assurance of data quality and quality control in medical registries [4]–[6]. These methods may include site visits, ongoing training programs, use of standardized definitions and regular audits of the data for completeness and consistency [4]–[6]. The importance, registries should probably monitor not only data quality but also associations and clinical predictions. In order to monitor data quality, we established a system to generate automated data quality control and prediction reports based on the statistical language R [51]. As our registry is an ongoing study, the results provided here are empirical examples from a limited number of patients. However, automated data quality control and prediction reports will be frequently updated and will be available under our data repository [57].

The demand for timely real-world data to support decision-making has driven the development of an increasing numbers of open data collections [17]–[20]. Adoption of open data policy is being encouraged not only by the U.S. government but globally by the editors of peer-reviewed journals [67]. Of the importance, open global databases are inherently necessary to accelerate the speed of evidence-based medicine and for an efficient, cost-effective healthcare system to improve the quality of patient care [1]–[8], [17]–[20]. Within our Open Data Collection protocol, socio-demographic, comorbidities and clinical characterization of patients undergoing pacemaker implantation will be publicly available in real time on a clouded-based repository following the concept of open data collection and under privacy, security and confidentiality policies (HIPAA) [25]. In addition to the data made available within clinicaltrials.gov, these variables will assist in the characterization of the study population for proper interpretation of published study results. The most important aspect of this approach is to foster a continuum between clinical care and clinical research leveraging the evidence development which may be successfully translated into better patient outcomes.

Using data derived from a randomized clinical trial is both a limitation and strength of our study. While randomized clinical trials are often conducted under high scientific methodological standards, their generalizability could be limited by including selected populations. On the other hand, the randomization of patients included in our registry will allow the comparison of long-term outcomes between different treatment alternatives, which is a key strength of this open registry collection. Implementation of other technology solutions such as integration with a platform for adverse events monitoring, protocols for data augmentation through natural language processing (NLP), open literature repositories connected to R Markdown files and protocols for enhance patients follow-up are future perspectives that will guide our next efforts. Finally, this registry can not only be used for the comparison of data within pacemaker patients but also as a source for comparison and benchmarking between different conditions within and between institutions. We believe that the framework proposed in this article can be a useful tool for creating high quality and interoperable medical registries.

## Supporting Information

Report S1 **Data quality report associated to the project “Pacemaker Registry – Open Data Collection.**
(HTML)

Table S1 **Pacemaker Registry Clinical Data Standards Elements.**
(DOCX)

Table S2 **Reimbursed values paid by Brazilian government for pacemaker implantation according to Brazilian States.**
(DOCX)

Table S3 **Cardiac Pacemaker Clinical Trials available at LinkedCT.**
(DOCX)
