# Correction: Glocal Clinical Registries: Pacemaker Registry Design and Implementation for Global and Local Integration – Methodology and Case Study

**DOI:** 10.1371/annotation/4df3845a-5099-4b10-b19a-0804cf201345

**Published:** 2013-10-23

**Authors:** Kátia Regina da Silva, Roberto Costa, Elizabeth Sartori Crevelari, Marianna Sobral Lacerda, Caio Marcos de Moraes Albertini, Martino Martinelli Filho, José Eduardo Santana, João Ricardo Nickenig Vissoci, Ricardo Pietrobon, Jacson V. Barros

An additional grant from CAPES Foundation, Ministry of Education of Brazil was incorrectly omitted from the Funding Statement. The Funding Statement should read: "This study was supported by CAPES Foundation, Ministry of Education of Brazil [Postdoctoral Fellowship Program 3928/11-0 and Foreign Visiting Professor Program 115/2012]. The funders had no role in study design, data collection and analysis, decision to publish, or preparation of the manuscript." 

