# Supplementary material for: Glocal Clinical Registries: Pacemaker Registry Design and Implementation for Global and Local Integration – Methodology and Case Study
Source: PLoS One. 2013 Jul 25;8(7):e71090. doi: 10.1371/journal.pone.0071090 (PMC3723676; doi:10.1371/journal.pone.0071090)
Supplement: Report S1 — Data quality report associated to the project “Pacemaker Registry – Open Data Collection. (HTML) [file pone.0071090.s001.html]

Data quality report associated to the project “Pacemaker Registry - Open Data Collection”


# Data quality report associated to the project “Pacemaker Registry - Open Data Collection”

Kátia Regina da Silva, RN PhD - katia.regina@incor.usp.br,
Roberto Costa, MD PhD, - rcosta@incor.usp.br
Elizabeth Sartori Crevelari MD - elizabethkaratebrasil@gmail.com
Marianna Sobral Lacerda, RN - sobralmarianna@gmail.com
Caio Marcos de Moraes Albertini, MD - email cmalbertini@gmail.com
Martino Martinelli Filho, MD PhD - email martino@incor.usp.br
Jose Eduardo Santana, MSc candidate jes@ic.ufal.br
Jacson Barros, MSc Jacsonv@ffm.br
Joao Ricardo Nickenig Vissoci, PhD candidate jnv4@duke.edu
Ricardo Pietrobon, MD, PhD rpietro@duke.edu

## Introduction

The main objective of this report is to provide a 24/7 snapshop at the data quality behind the Pacemaker Registry - Open Data Collection project. This includes not only the original data set collected under Dr. Katia Regina da Silva, Brazil, but also any additional data sets brought in from other registries, prospective studies, as well as Linked Open Data sources.

Methodological details about the study can be found at Pacemaker Registry - Open Data Collection.

## Data Description

Below is a full list of variables along with their class (variable type according to the R language) and alternative responses. Most of the variable names should be intuitive, but a full dictionary is also be found at Pacemaker Registry - Open Data Collection.

A description of missing values is provided in the Figures below the descriptions, where missing values are presented in yellow.

```
## Miss1 
## 
##  10  Variables      27  Observations
## ---------------------------------------------------------------------------
## date_enrolled 
##       n missing  unique 
##      27       0      25 
## 
## lowest : 1/3/13   1/7/13   1/9/13   10/10/12 10/25/12
## highest: 7/30/12  7/6/12   8/16/12  8/8/12   9/20/12  
## ---------------------------------------------------------------------------
## sex 
##       n missing  unique     Sum    Mean 
##      27       0       2      12  0.4444 
## ---------------------------------------------------------------------------
## dob 
##       n missing  unique 
##      27       0      27 
## 
## lowest : 1/1/51   1/15/46  1/27/45  1/9/53   10/15/53
## highest: 7/26/46  7/6/38   8/11/26  8/25/31  9/25/51  
## ---------------------------------------------------------------------------
## age_enrollment 
##       n missing  unique    Mean     .05     .10     .25     .50     .75 
##      27       0      21   65.96    51.3    53.2    60.0    67.0    70.5 
##     .90     .95 
##    79.8    81.7 
## 
## lowest : 41 51 52 54 56, highest: 75 79 81 82 85 
## ---------------------------------------------------------------------------
## marital_status 
##       n missing  unique    Mean 
##      27       0       4       2 
## 
## 1 (7, 26%), 2 (15, 56%), 3 (3, 11%), 4 (2, 7%) 
## ---------------------------------------------------------------------------
## have_children 
##       n missing  unique     Sum    Mean 
##      26       1       2      20  0.7692 
## ---------------------------------------------------------------------------
## num_children 
##       n missing  unique    Mean 
##      20       7       6     3.8 
## 
##           1  2  3  4  5  7
## Frequency 1  5  4  3  4  3
## %         5 25 20 15 20 15
## ---------------------------------------------------------------------------
## race 
##       n missing  unique    Mean 
##      27       0       3  0.2963 
## 
## 0 (22, 81%), 1 (2, 7%), 2 (3, 11%) 
## ---------------------------------------------------------------------------
## ethnicity 
##       n missing  unique    Mean 
##      27       0       2 0.07407 
## 
## 0 (26, 96%), 2 (1, 4%) 
## ---------------------------------------------------------------------------
## professional_category 
##       n missing  unique    Mean 
##      27       0       7   15.67 
## 
##           2 3 13 14 15 21 28
## Frequency 2 2  1 10  1  9  2
## %         7 7  4 37  4 33  7
## ---------------------------------------------------------------------------
```

```
##       V1 V3 V4 V5 V6 V7 V8 V9 V10 V11 Total
## 1      1  1  1  1  1  1  1  1   1   1    20
## 2      1  1  1  1  1  1  0  1   1   1     6
## 3      1  1  1  1  1  0  0  1   1   1     1
## Total  0  0  0  0  0  1  7  0   0   0    27
```

```
## Miss2 
## 
##  28  Variables      27  Observations
## ---------------------------------------------------------------------------
## date_baseline_evaluation 
##       n missing  unique 
##      27       0      25 
## 
## lowest : 1/3/13   1/7/13   1/9/13   10/10/12 10/25/12
## highest: 7/30/12  7/6/12   8/16/12  8/8/12   9/20/12  
## ---------------------------------------------------------------------------
## functional_class_basal 
##       n missing  unique    Mean 
##      27       0       4   2.111 
## 
## 1 (6, 22%), 2 (13, 48%), 3 (7, 26%), 4 (1, 4%) 
## ---------------------------------------------------------------------------
## underlying_heart_disease 
##       n missing  unique    Mean 
##      27       0       5   8.778 
## 
##           2  3 5 6 12
## Frequency 2  6 1 1 17
## %         7 22 4 4 63
## ---------------------------------------------------------------------------
## comorbid 
##       n missing  unique     Sum    Mean 
##      27       0       2      26   0.963 
## ---------------------------------------------------------------------------
## allergic_iodine 
##       n missing  unique    Mean 
##      27       0       1       2 
## ---------------------------------------------------------------------------
## date_preoperative_ecg 
##       n missing  unique 
##      27       0      24 
## 
## lowest : 1/2/13   1/4/13   10/24/12 10/29/12 11/1/12 
## highest: 8/1/13   8/15/12  8/7/12   9/18/12  9/30/12  
## ---------------------------------------------------------------------------
## ecg_rhythm_preop 
##       n missing  unique    Mean 
##      27       0       4   10.85 
## 
## 4 (2, 7%), 10 (6, 22%), 11 (3, 11%), 12 (16, 59%) 
## ---------------------------------------------------------------------------
## atrial_heart_rate_basal 
##       n missing  unique    Mean     .05     .10     .25     .50     .75 
##      25       2      17   78.56    58.4    60.0    69.0    76.0    84.0 
##     .90     .95 
##    90.0   102.0 
## 
##           55 58 60 64 69 70 72 75 76 78 79 80 84 85 90 105 160
## Frequency  1  1  3  1  1  1  1  3  2  2  1  1  1  1  3   1   1
## %          4  4 12  4  4  4  4 12  8  8  4  4  4  4 12   4   4
## ---------------------------------------------------------------------------
## ventric_heart_rate_basal 
##       n missing  unique 
##      27       0      17 
## 
##           10 28 30 31 33 35 36 37 38 39 40 41 42 46 47 58 sem escape
## Frequency  1  1  1  2  1  1  1  2  3  5  3  1  1  1  1  1          1
## %          4  4  4  7  4  4  4  7 11 19 11  4  4  4  4  4          4
## ---------------------------------------------------------------------------
## p_duration_basal 
##       n missing  unique    Mean 
##      26       1       3   95.38 
## 
## 80 (10, 38%), 100 (12, 46%), 120 (4, 15%) 
## ---------------------------------------------------------------------------
## ecg_pri_obtainable 
##       n missing  unique    Mean 
##      27       0       1       0 
## ---------------------------------------------------------------------------
## qrs_duration_basal 
##       n missing  unique    Mean     .05     .10     .25     .50     .75 
##      27       0      17   130.9    80.9    97.4   121.0   138.0   142.0 
##     .90     .95 
##   151.6   158.2 
## 
##           79 80 83 107 119 120 122 126 129 134 138 140 141 142 150 154 160
## Frequency  1  1  1   1   1   2   1   1   2   2   1   1   2   4   3   1   2
## %          4  4  4   4   4   7   4   4   7   7   4   4   7  15  11   4   7
## ---------------------------------------------------------------------------
## qrs_axis_basal 
##       n missing  unique    Mean     .05     .10     .25     .50     .75 
##      27       0      27   29.85   -88.8   -84.8   -63.5    15.0    98.5 
##     .90     .95 
##   230.0   249.8 
## 
## lowest : -120  -90  -86  -84  -78, highest:  133  228  233  257  258 
## ---------------------------------------------------------------------------
## xray_date 
##       n missing  unique 
##      27       0      26 
## 
## lowest : 1/2/13   1/4/13   1/9/13   10/19/12 10/29/12
## highest: 7/30/12  7/6/12   8/15/12  8/8/12   9/18/12  
## ---------------------------------------------------------------------------
## date_lab_tests_basal 
##       n missing  unique 
##      27       0      24 
## 
## lowest : 1/3/13   1/7/13   1/9/13   10/10/12 10/24/12
## highest: 7/30/12  7/6/12   8/15/12  8/7/12   9/18/12  
## ---------------------------------------------------------------------------
## hemoglobin_basal 
##       n missing  unique    Mean     .05     .10     .25     .50     .75 
##      27       0      21   14.37   11.19   12.28   13.10   14.50   16.00 
##     .90     .95 
##   17.00   17.00 
## 
## lowest : 10.2 10.8 12.1 12.4 12.7, highest: 15.2 16.0 16.3 17.0 17.5 
## ---------------------------------------------------------------------------
## hematocrit_basal 
##       n missing  unique    Mean     .05     .10     .25     .50     .75 
##      27       0      15   43.66   34.90   37.54   40.00   44.00   49.50 
##     .90     .95 
##   51.00   51.00 
## 
##           32 34 37 37.9 38 39 40 41 42 44 45 49 50 51 53
## Frequency  1  1  1    1  1  1  3  2  2  1  5  1  2  4  1
## %          4  4  4    4  4  4 11  7  7  4 19  4  7 15  4
## ---------------------------------------------------------------------------
## platelets_basal 
##       n missing  unique    Mean     .05     .10     .25     .50     .75 
##      27       0      24  228333  135100  160000  179000  216000  250000 
##     .90     .95 
##  338800  409800 
## 
## lowest : 109000 127000 154000 164000 171000
## highest: 265000 298000 400000 414000 449000 
## ---------------------------------------------------------------------------
## leukocytes_basal 
##       n missing  unique    Mean     .05     .10     .25     .50     .75 
##      27       0      27    8167    4936    5576    6510    7740    9645 
##     .90     .95 
##   10642   11407 
## 
## lowest :  4240  4870  5090  5900  6270
## highest: 10080 10230 11260 11470 16820 
## ---------------------------------------------------------------------------
## urea_basal 
##       n missing  unique    Mean     .05     .10     .25     .50     .75 
##      26       1      19    44.5   22.75   31.00   35.75   40.00   50.00 
##     .90     .95 
##   62.00   82.75 
## 
##           11 20 31 33 35 38 39 40 43 44 45 48 50 53 57 60 64 89 90
## Frequency  1  1  3  1  1  3  2  2  1  1  1  1  2  1  1  1  1  1  1
## %          4  4 12  4  4 12  8  8  4  4  4  4  8  4  4  4  4  4  4
## ---------------------------------------------------------------------------
## creatinine_basal 
##       n missing  unique    Mean     .05     .10     .25     .50     .75 
##      27       0      25   1.143   0.813   0.826   0.905   1.020   1.300 
##     .90     .95 
##   1.504   1.638 
## 
## lowest : 0.77 0.81 0.82 0.83 0.84, highest: 1.42 1.48 1.54 1.68 2.43 
## ---------------------------------------------------------------------------
## sodium_basal 
##       n missing  unique    Mean 
##      26       1       9   140.2 
## 
##           135 136 138 139 140 141 142 143 144
## Frequency   1   2   2   7   3   3   1   4   3
## %           4   8   8  27  12  12   4  15  12
## ---------------------------------------------------------------------------
## potassium_basal 
##       n missing  unique    Mean     .05     .10     .25     .50     .75 
##      27       0      12   4.385    3.93    4.00    4.10    4.40    4.55 
##     .90     .95 
##    4.78    5.11 
## 
##           3.9  4 4.1 4.2 4.3 4.4 4.5 4.6 4.7 4.9 5.2 5.3
## Frequency   2  3   5   1   2   1   6   3   1   1   1   1
## %           7 11  19   4   7   4  22  11   4   4   4   4
## ---------------------------------------------------------------------------
## prothrombin_time_basal 
##       n missing  unique    Mean     .05     .10     .25     .50     .75 
##      25       2      19   14.78   13.34   13.66   14.10   14.80   15.00 
##     .90     .95 
##   15.86   17.26 
## 
##           13.2 13.3 13.5 13.9 14 14.1 14.2 14.3 14.4 14.5 14.8 14.9 15
## Frequency    1    1    1    2  1    1    2    1    1    1    3    2  2
## %            4    4    4    8  4    4    8    4    4    4   12    8  8
##           15.2 15.7 15.8 15.9 17.6 17.7
## Frequency    1    1    1    1    1    1
## %            4    4    4    4    4    4
## ---------------------------------------------------------------------------
## prothrombin_activity_basal 
##       n missing  unique    Mean     .05     .10     .25     .50     .75 
##      25       2      23   89.06   61.58   67.78   84.50   91.50   98.70 
##     .90     .95 
##  104.08  109.16 
## 
## lowest :  36.9  61.2  63.1  74.8  76.5
## highest: 101.3 102.7 105.0 110.2 111.8 
## ---------------------------------------------------------------------------
## aptt_basal 
##       n missing  unique    Mean     .05     .10     .25     .50     .75 
##      25       2      22   31.34   24.20   24.52   26.10   29.00   32.50 
##     .90     .95 
##   33.84   37.20 
## 
## lowest : 22.3 24.2 25.0 25.7 25.9, highest: 33.1 33.6 34.0 38.0 85.6 
## ---------------------------------------------------------------------------
## inr_basal 
##       n missing  unique    Mean 
##      25       2       5   1.072 
## 
##           0.9  1 1.1 1.2 1.3
## Frequency   1 11   9   2   2
## %           4 44  36   8   8
## ---------------------------------------------------------------------------
## 
## Variables with all observations missing:
## 
## [1] pri_duration_basal
```

```
##       V12 V13 V14 V15 V16 V17 V18 V19 V20 V21 V22 V23 V24 V25 V26 V27 V28
## 1       1   1   1   1   1   1   1   1   1   1   1   0   1   1   1   1   1
## 2       1   1   1   1   1   1   1   1   1   1   1   0   1   1   1   1   1
## 3       1   1   1   1   1   1   1   1   1   1   1   0   1   1   1   1   1
## 4       1   1   1   1   1   1   1   1   1   1   1   0   1   1   1   1   1
## 5       1   1   1   1   1   1   1   0   1   1   1   0   1   1   1   1   1
## 6       1   1   1   1   1   1   1   0   1   0   1   0   1   1   1   1   1
## Total   0   0   0   0   0   0   0   2   0   1   0  27   0   0   0   0   0
##       V29 V30 V31 V32 V33 V34 V35 V36 V37 V38 V39 Total
## 1       1   1   1   1   1   1   1   1   1   1   1    22
## 2       1   1   1   1   1   1   1   0   0   0   0     1
## 3       1   1   1   1   1   0   1   0   0   0   0     1
## 4       1   1   1   0   1   1   1   1   1   1   1     1
## 5       1   1   1   1   1   1   1   1   1   1   1     1
## 6       1   1   1   1   1   1   1   1   1   1   1     1
## Total   0   0   0   1   0   1   0   2   2   2   2    27
```

```
## Miss3 
## 
##  23  Variables      27  Observations
## ---------------------------------------------------------------------------
## randomization 
##       n missing  unique    Mean 
##      27       0       2   1.481 
## 
## 1 (14, 52%), 2 (13, 48%) 
## ---------------------------------------------------------------------------
## date_pm_implantation 
##       n missing  unique 
##      27       0      24 
## 
## lowest : 1/10/13  1/4/13   1/8/13   10/15/12 10/26/12
## highest: 7/31/12  7/6/12   8/10/12  8/17/12  9/21/12  
## ---------------------------------------------------------------------------
## temporary_pm 
##       n missing  unique     Sum    Mean 
##      27       0       2      17  0.6296 
## ---------------------------------------------------------------------------
## temporary_pm_date 
##       n missing  unique 
##      27       0      18 
## 
##              1/9/13 11/1/12 11/12/12 11/19/12 11/23/12 11/5/12 12/10/12
## Frequency 10      1       1        1        1        1       1        1
## %         37      4       4        4        4        4       4        4
##           12/3/12 12/8/12 6/24/12 6/4/12 6/8/12 7/20/12 7/21/12 7/26/12
## Frequency       1       1       1      1      1       1       1       1
## %               4       4       4      4      4       4       4       4
##           7/29/12 8/16/12
## Frequency       1       1
## %               4       4
## ---------------------------------------------------------------------------
## days_temporary_pacing 
##       n missing  unique    Mean 
##      17      10       9   5.529 
## 
##            1  2  3  4  5 6 7 9 32
## Frequency  2  3  2  4  2 1 1 1  1
## %         12 18 12 24 12 6 6 6  6
## ---------------------------------------------------------------------------
## temporary_pm_venous_access 
##       n missing  unique    Mean 
##      17      10       1       1 
## ---------------------------------------------------------------------------
## type_anesthesia 
##       n missing  unique    Mean 
##      27       0       2   2.481 
## 
## 2 (14, 52%), 3 (13, 48%) 
## ---------------------------------------------------------------------------
## procedure_performed 
##       n missing  unique    Mean 
##      27       0       2   1.963 
## 
## 1 (1, 4%), 2 (26, 96%) 
## ---------------------------------------------------------------------------
## time_entry_operation_room 
##       n missing  unique 
##      27       0      26 
## 
## lowest : 1/10/13 14:20  1/4/13 10:45   1/4/13 7:20    1/8/13 13:30   10/15/12 10:20
## highest: 7/31/12 12:40  7/6/12 14:00   8/10/12 13:30  8/17/12 7:20   9/21/12 11:21  
## ---------------------------------------------------------------------------
## incision_time 
##       n missing  unique 
##      27       0      27 
## 
## lowest : 1/10/13 15:16  1/4/13 11:30   1/4/13 8:30    1/8/13 14:00   10/15/12 10:58
## highest: 7/31/12 13:25  7/6/12 14:50   8/10/12 14:20  8/17/12 8:36   9/21/12 12:01  
## ---------------------------------------------------------------------------
## vascular_access_time 
##       n missing  unique 
##      27       0      27 
## 
## lowest : 1/10/13 15:26  1/4/13 11:38   1/4/13 8:35    1/8/13 14:12   10/15/12 11:03
## highest: 7/31/12 13:30  7/6/12 15:00   8/10/12 14:45  8/17/12 9:02   9/21/12 12:12  
## ---------------------------------------------------------------------------
## ventr_lead_placement_time 
##       n missing  unique 
##      27       0      27 
## 
## lowest : 1/10/13 15:32  1/4/13 12:07   1/4/13 8:39    1/8/13 14:17   10/15/12 11:50
## highest: 7/31/12 14:12  7/6/12 15:35   8/10/12 15:24  8/17/12 10:03  9/21/12 12:16  
## ---------------------------------------------------------------------------
## skin_closure_time 
##       n missing  unique 
##      27       0      27 
## 
## lowest : 1/10/13 16:02  1/4/13 12:52   1/4/13 9:09    1/8/13 14:56   10/15/12 12:38
## highest: 7/31/12 14:45  7/6/12 16:13   8/10/12 16:45  8/17/12 11:12  9/21/12 12:59  
## ---------------------------------------------------------------------------
## total_fluoroscopy_time 
##       n missing  unique 
##      27       0      14 
## 
##             1.57 14.26 15 20 24 3.06 4 5 5.09 5.3 7.04 7.53 not available
## Frequency 1    1     1  1  1  1    1 1 1    1   1    1    1            14
## %         4    4     4  4  4  4    4 4 4    4   4    4    4            52
## ---------------------------------------------------------------------------
## total_lead_positioning 
##       n missing  unique    Mean     .05     .10     .25     .50     .75 
##      27       0      21   29.48     2.3     3.0     4.5    12.0    43.5 
##     .90     .95 
##    69.8    97.0 
## 
## lowest :   2   3   4   5   6, highest:  60  61  83 103 119 
## ---------------------------------------------------------------------------
## total_procedure_time 
##       n missing  unique    Mean     .05     .10     .25     .50     .75 
##      27       0      26   83.96    38.3    42.6    56.5    69.0   111.0 
##     .90     .95 
##   149.4   156.0 
## 
## lowest :  34  38  39  45  46, highest: 125 131 145 156 167 
## ---------------------------------------------------------------------------
## pulse_generator_model 
##       n missing  unique    Mean 
##      27       0       2   1.963 
## 
## 1 (1, 4%), 2 (26, 96%) 
## ---------------------------------------------------------------------------
## pulse_generator_sn 
##       n missing  unique 
##      27       0      27 
## 
## lowest : NWA613090S NWA628448S NWB906516S NWB925982S NWB926270S
## highest: NWD630455S NWD630457S NWD631220S NWD631222S NWP614272S 
## ---------------------------------------------------------------------------
## pulse_generator_pocket 
##       n missing  unique    Mean 
##      27       0       2   1.037 
## 
## 1 (26, 96%), 2 (1, 4%) 
## ---------------------------------------------------------------------------
## pulse_generator_site 
##       n missing  unique    Mean 
##      27       0       1       2 
## ---------------------------------------------------------------------------
## crossover 
##       n missing  unique     Sum    Mean 
##      27       0       2       3  0.1111 
## ---------------------------------------------------------------------------
## intraop_complications 
##       n missing  unique     Sum    Mean 
##      27       0       2       2 0.07407 
## ---------------------------------------------------------------------------
## length_hospital_stay 
##       n missing  unique    Mean     .05     .10     .25     .50     .75 
##      24       3      10   8.042    3.15    4.00    4.75    6.00    9.00 
##     .90     .95 
##   13.40   17.40 
## 
##           3  4  5  6 8  9 12 14 18 33
## Frequency 2  4  3  6 2  3  1  1  1  1
## %         8 17 12 25 8 12  4  4  4  4
## ---------------------------------------------------------------------------
```

```
##       V40 V41 V42 V43 V44 V45 V46 V47 V48 V49 V50 V51 V52 V53 V54 V55 V56
## 1       1   1   1   1   1   1   1   1   1   1   1   1   1   1   1   1   1
## 2       1   1   1   1   1   1   1   1   1   1   1   1   1   1   1   1   1
## 3       1   1   1   1   0   0   1   1   1   1   1   1   1   1   1   1   1
## 4       1   1   1   1   0   0   1   1   1   1   1   1   1   1   1   1   1
## Total   0   0   0   0  10  10   0   0   0   0   0   0   0   0   0   0   0
##       V57 V58 V59 V60 V61 V62 Total
## 1       1   1   1   1   1   1    16
## 2       1   1   1   1   1   0     1
## 3       1   1   1   1   1   1     8
## 4       1   1   1   1   1   0     2
## Total   0   0   0   0   0   3    27
```

```
## Miss4 
## 
##  28  Variables      27  Observations
## ---------------------------------------------------------------------------
## ra_lead_model 
##       n missing  unique 
##      27       0       3 
## 
##  (1, 4%), 5076 (1, 4%), 5076 52 (25, 93%) 
## ---------------------------------------------------------------------------
## ra_lead_sn 
##       n missing  unique 
##      27       0      27 
## 
## lowest :            PJN2677802 PJN2715912 PJN2767649 PJN2767656
## highest: PJN2876090 PJN2884311 PJN2884312 PJN2884313 PJN2891774 
## ---------------------------------------------------------------------------
## ra_lead_venous_access 
##       n missing  unique    Mean 
##      26       1       3   3.731 
## 
## 2 (3, 12%), 3 (1, 4%), 4 (22, 85%) 
## ---------------------------------------------------------------------------
## ra_lead_positioning 
##       n missing  unique    Mean 
##      25       2       3    2.04 
## 
## 1 (1, 4%), 2 (23, 92%), 4 (1, 4%) 
## ---------------------------------------------------------------------------
## ra_lead_threshold_uni 
##       n missing  unique    Mean 
##      26       1       9     0.7 
## 
##           0.3 0.4 0.5 0.6 0.7 0.8  1 1.1 1.3
## Frequency   3   2   4   6   1   3  3   2   2
## %          12   8  15  23   4  12 12   8   8
## ---------------------------------------------------------------------------
## ra_lead_threshold_bi 
##       n missing  unique    Mean     .05     .10     .25     .50     .75 
##      26       1      12     0.8   0.400   0.450   0.525   0.750   1.000 
##     .90     .95 
##   1.250   1.375 
## 
##           0.4 0.5 0.6 0.7 0.8 0.9 1 1.1 1.2 1.3 1.4 1.5
## Frequency   3   4   4   2   3   2 2   2   1   1   1   1
## %          12  15  15   8  12   8 8   8   4   4   4   4
## ---------------------------------------------------------------------------
## ra_lead_impedance_uni 
##       n missing  unique    Mean     .05     .10     .25     .50     .75 
##      26       1      24     675   474.2   477.5   549.2   586.5   630.8 
##     .90     .95 
##   825.0  1430.0 
## 
## lowest :  450  474  475  480  503, highest:  717  730  920 1600 1800 
## ---------------------------------------------------------------------------
## ra_lead_impedance_bi 
##       n missing  unique    Mean     .05     .10     .25     .50     .75 
##      26       1      22   789.2   558.2   590.0   640.0   690.0   813.0 
##     .90     .95 
##   921.5  1507.8 
## 
## lowest :  520  551  580  600  607, highest:  875  912  931 1700 2000 
## ---------------------------------------------------------------------------
## ra_lead_sense_p_uni 
##       n missing  unique    Mean     .05     .10     .25     .50     .75 
##      26       1      20   2.742   1.425   1.550   1.850   2.650   3.150 
##     .90     .95 
##   4.250   4.875 
## 
## lowest : 1.0 1.4 1.5 1.6 1.7, highest: 3.9 4.0 4.5 5.0 5.5 
## ---------------------------------------------------------------------------
## ra_lead_sense_p_bi 
##       n missing  unique    Mean     .05     .10     .25     .50     .75 
##      26       1      18   3.127   1.325   1.750   2.500   3.000   3.875 
##     .90     .95 
##   4.800   5.000 
## 
##           1 1.2 1.7 1.8 2 2.5 2.6 2.8 3 3.5 3.6 3.7 3.8 3.9 4 4.5 4.6  5
## Frequency 1   1   1   1 2   3   2   1 2   2   1   1   1   1 1   1   1  3
## %         4   4   4   4 8  12   8   4 8   8   4   4   4   4 4   4   4 12
## ---------------------------------------------------------------------------
## rv_lead_model 
##       n missing  unique 
##      27       0       3 
## 
##  (10, 37%), 5076 (1, 4%), 5076 58 (16, 59%) 
## ---------------------------------------------------------------------------
## rv_lead_sn 
##       n missing  unique 
##      27       0      18 
## 
##              PJN2613929 PJN2700285 PJN2700290 PJN2700300 PJN2792862
## Frequency 10          1          1          1          1          1
## %         37          4          4          4          4          4
##           PJN2793417 PJN2796271 PJN2826569 PJN2875378 PJN2875632
## Frequency          1          1          1          1          1
## %                  4          4          4          4          4
##           PJN2891751 PJN2891752 PJN2891774 PJN2891783 PJN2895644
## Frequency          1          1          1          1          1
## %                  4          4          4          4          4
##           PJN2908118 PJN2908132
## Frequency          1          1
## %                  4          4
## ---------------------------------------------------------------------------
## rv_lead_venous_access 
##       n missing  unique    Mean 
##      17      10       3   3.588 
## 
## 1 (1, 6%), 2 (2, 12%), 4 (14, 82%) 
## ---------------------------------------------------------------------------
## rv_lead_position 
##       n missing  unique    Mean 
##      17      10       2   1.706 
## 
## 1 (5, 29%), 2 (12, 71%) 
## ---------------------------------------------------------------------------
## rv_lead_threshold 
##       n missing  unique    Mean     .05     .10     .25     .50     .75 
##      17      10      11  0.7706    0.30    0.36    0.50    0.60    1.00 
##     .90     .95 
##    1.40    1.42 
## 
##           0.3 0.4 0.5 0.6 0.7 0.8 0.9 1 1.2 1.4 1.5
## Frequency   2   2   2   3   1   1   1 1   1   2   1
## %          12  12  12  18   6   6   6 6   6  12   6
## ---------------------------------------------------------------------------
## rv_lead_threshold_bi 
##       n missing  unique    Mean     .05     .10     .25     .50     .75 
##      17      10      12  0.8353    0.28    0.36    0.60    0.70    1.00 
##     .90     .95 
##    1.54    1.60 
## 
##           0.2 0.3 0.4 0.5 0.6 0.7 0.8 0.9 1 1.3 1.5 1.6
## Frequency   1   1   1   1   1   5   1   1 1   1   1   2
## %           6   6   6   6   6  29   6   6 6   6   6  12
## ---------------------------------------------------------------------------
## rv_lead_impedance_uni 
##       n missing  unique    Mean     .05     .10     .25     .50     .75 
##      17      10      14   857.8   580.0   605.6   650.0   830.0  1000.0 
##     .90     .95 
##  1000.0  1140.0 
## 
##           564 584 620 640 650 670 717 818 830 900 920 970 1000 1700
## Frequency   1   1   1   1   1   1   1   1   1   1   1   1    4    1
## %           6   6   6   6   6   6   6   6   6   6   6   6   24    6
## ---------------------------------------------------------------------------
## rv_lead_impedance_bi 
##       n missing  unique    Mean     .05     .10     .25     .50     .75 
##      17      10      15   978.5   657.2   671.8   720.0   930.0  1100.0 
##     .90     .95 
##  1224.0  1388.0 
## 
##           630 664 677 714 720 738 920 930 1000 1071 1080 1100 1200 1260
## Frequency   1   1   1   1   1   1   1   2    1    1    1    2    1    1
## %           6   6   6   6   6   6   6  12    6    6    6   12    6    6
##           1900
## Frequency    1
## %            6
## ---------------------------------------------------------------------------
## rv_lead_sense_r_uni 
##       n missing  unique    Mean 
##      11      16       8   8.018 
## 
##           3.7 4.5 5.5  6  7 9 16 18
## Frequency   1   1   2  2  2 1  1  1
## %           9   9  18 18 18 9  9  9
## ---------------------------------------------------------------------------
## rv_lead_sense_r_bi 
##       n missing  unique    Mean     .05     .10     .25     .50     .75 
##      10      17      10    9.38   3.840   4.380   6.625   9.000  12.375 
##     .90     .95 
##  13.400  15.200 
## 
##           3.3 4.5 6.5  7  8 10 12 12.5 13 17
## Frequency   1   1   1  1  1  1  1    1  1  1
## %          10  10  10 10 10 10 10   10 10 10
## ---------------------------------------------------------------------------
## lv_lead_model 
##       n missing  unique 
##      27       0       2 
## 
##  (17, 63%), 4195 88 (10, 37%) 
## ---------------------------------------------------------------------------
## lv_lead_sn 
##       n missing  unique 
##      27       0      11 
## 
##              AAD055343V AAD055347V AAD055349V AAD055350V AAD061001V
## Frequency 17          1          1          1          1          1
## %         63          4          4          4          4          4
##           AAD066414V AAD066417V AAD066672V AAD070731V AAD51499V
## Frequency          1          1          1          1         1
## %                  4          4          4          4         4
## ---------------------------------------------------------------------------
## lv_lead_venous_access 
##       n missing  unique    Mean 
##      10      17       2     3.6 
## 
## 2 (2, 20%), 4 (8, 80%) 
## ---------------------------------------------------------------------------
## lv_lead_pacing_site 
##       n missing  unique    Mean 
##       9      18       2   2.222 
## 
## 2 (7, 78%), 3 (2, 22%) 
## ---------------------------------------------------------------------------
## lv_lead_threshold_uni 
##       n missing  unique    Mean 
##      10      17       9    1.41 
## 
##           0.3 0.5 0.7 0.8 1.1 1.2 1.8 3.3 3.6
## Frequency   1   1   1   2   1   1   1   1   1
## %          10  10  10  20  10  10  10  10  10
## ---------------------------------------------------------------------------
## lv_lead_impedance_uni 
##       n missing  unique    Mean     .05     .10     .25     .50     .75 
##      10      17      10   782.8   545.0   590.0   639.0   748.0   882.5 
##     .90     .95 
##  1020.0  1110.0 
## 
##           500 600 637 645 674 822 860 890 1000 1200
## Frequency   1   1   1   1   1   1   1   1    1    1
## %          10  10  10  10  10  10  10  10   10   10
## ---------------------------------------------------------------------------
## lv_lead_sense_r_uni 
##       n missing  unique    Mean 
##       7      20       7   8.786 
## 
##            4 4.5  5  8  9 13 18
## Frequency  1   1  1  1  1  1  1
## %         14  14 14 14 14 14 14
## ---------------------------------------------------------------------------
## escape_rhythm 
##       n missing  unique     Sum    Mean 
##      27       0       2      11  0.4074 
## ---------------------------------------------------------------------------
```

```
##       V63 V64 V65 V66 V67 V68 V69 V70 V71 V72 V73 V74 V75 V76 V77 V78 V79
## 1       1   1   1   1   1   1   1   1   1   1   1   1   1   1   1   1   1
## 2       1   1   1   1   1   1   1   1   1   1   1   1   1   1   1   1   1
## 3       1   1   1   1   1   1   1   1   1   1   1   1   1   1   1   1   1
## 4       1   1   1   1   1   1   1   1   1   1   1   1   0   0   0   0   0
## 5       1   1   1   1   1   1   1   1   1   1   1   1   0   0   0   0   0
## 6       1   1   1   1   1   1   1   1   1   1   1   1   0   0   0   0   0
## 7       1   1   1   0   1   1   1   1   1   1   1   1   1   1   1   1   1
## 8       1   1   0   0   0   0   0   0   0   0   1   1   1   1   1   1   1
## Total   0   0   1   2   1   1   1   1   1   1   0   0  10  10  10  10  10
##       V80 V81 V82 V83 V84 V85 V86 V87 V88 V89 V90 Total
## 1       1   1   1   1   1   0   0   0   0   0   1     8
## 2       1   1   0   1   1   0   0   0   0   0   1     1
## 3       1   0   0   1   1   0   0   0   0   0   1     6
## 4       0   0   0   1   1   1   1   1   1   1   1     6
## 5       0   0   0   1   1   1   1   1   1   0   1     3
## 6       0   0   0   1   1   1   0   1   1   1   1     1
## 7       1   1   1   1   1   0   0   0   0   0   1     1
## 8       1   1   1   1   1   0   0   0   0   0   1     1
## Total  10  16  17   0   0  17  18  17  17  20   0    27
```

## Data Quality

Below is a series of graph representations regarding data quality control. Firste set o graphs allow to evaluate trends, cycles, and shifts as data quality indicator. These graphs were created with the qcc package from R Language Open Software.

Another example using a different continuous variable (Lenght of hospital stay)

Also, OC Curves allow to evaluete power of the data quality control process. However, since we are working to control data quality for dataset with different temporal sample sizes, OC plots were not able to be created.

For categorical variables (e.g. randomization or underlying heart disease), we used data quality control charts for proportions over a group of subjects.

In order to monitor for possible data fabrication, we created graphics comparing single digit distribution against Benford's Law or first-digit law. Briefly, this law refers to the frequency of digits in real-life sources of data. For example, digit 1 occurs as the first digit in close to 30% of all numbers, while a digit such as 9 only occurs in about 5% of the time. In our graphics, we plot the expected versus the actual digit distribution.

## Data Association

Figures regarding associations between variables from the Glocal Registry project projetc are presente below. First set of figures show the the comparison of numeric variables from our dataset between subjects devided by randomization.

Data association and prediction plots are generated based on boxplots for reports on numeric data and association plots for categorical data. Two examples are shown. The first is a set of boxplots (a) presenting the comparison of Time of Pacemaker Implantation, Duration of the Procedure and Length of Hospital Stay between randomization groups (left and right ventricle). The second (b) one is an Cohen-Friendly association plot showing the proportion of complications between randomization groups. This plot shows the distribution of the outcome in a contingency table, represented by the rectangles. Red colored boxes represent a cell where the observed frequency is higher than the expected while the black figure shows the opposite, also the area of the boxes indicate the size of the difference.

Below is an application of the MINE (maximal Information-based Nonparametric Exploration) algorithm from the Science article Detecting Novel Association in Large Data Sets. See also http://www.exploredata.net/. This application was used to explore the associations between variables inside the dataset. Variables with highe values of linear relation (MIC) as well as the analysis of non-lnear associations (MAS) are shown in figures below.

After analyzing MINE results, we took the higher MIC and non-linear relations indicatores to plot in scatter plots. First set of plots show pairs of relation with MIC higher than 0.4, also clustered by randomization value.

Second set of plots show pairs of relation with MIC non-linear higher than 0.4, also clustered by randomization value. This allows one to observe non-linear associations.
