# Supplementary material for: Glocal Clinical Registries: Pacemaker Registry Design and Implementation for Global and Local Integration – Methodology and Case Study
Source: PLoS One. 2013 Jul 25;8(7):e71090. doi: 10.1371/journal.pone.0071090 (PMC3723676; doi:10.1371/journal.pone.0071090)
Supplement: Table S1 — Pacemaker Registry Clinical Data Standards Elements. (DOCX) [file pone.0071090.s002.docx]

| **Variable Class** | **Data Standards Elements** | **Source of Standardization** |
| --- | --- | --- |
| Patient Identifiers | Patient ID | NCI Thesaurus [42,43] |
| Patient Demographics | Date of birth, gender, race, ethnicity, address, insurance payer, presentation to healthcare facility | NCI Thesaurus, ACC/AHA [28-33] |
| Patient History | Presentations associated with arrhythmia, arrhythmia history, specific ECG patterns, NYHA functional classification, etiology, underlying heart disease, comorbid conditions, history of cardiovascular disease, history of non-cardiovascular diseases | ACC/AHA [28,29] |
| Laboratory Tests | Hemoglobin, hematocrit, platelet count, blood urea nitrogen, serum creatinine, potassium, sodium, calcium, magnesium, glucose, total cholesterol, INR | ACC/AHA [28-33] |
| Specific Electrocardiogram Patterns | Sinus bradycardia, sinus pause, brady-tachycardia syndrome, atrial fibrillation with slow ventricular response, atrial flutter with slow ventricular response, junctional rhythm, first-degree AV block, second-degree AV block - Mobitz I, second-degree AV block - Mobitz II, second-degree AV block - Type 2:1, third degree AV block, right bundle branch block, left bundle branch block, left anterior fascicular block, left posterior fascicular block, incomplete right bundle branch block | ACC/AHA [29,30], Trials [34-37] |
| Chest Radiography | Cardiac silhouette*, cardiothoracic ratio*, lung fields without alterations*, presence of pulmonary edema or pulmonary congestion | ACC/AHA [32], *data elements under development |
| Echocardiography before and after Pacemaker Implantation | Left ventricular end systolic volume, left ventricular end diastolic volume, left ventricular end systolic dimension, left ventricular end diastolic dimension, left ventricular ejection fraction (Simpson), left ventricular ejection fraction (Teichholz), left ventricular mass, hypokinesia, akinesia, valve disease, aortic root (M-mode), septal-to-posterior wall motion delay (M-mode), dyssynchrony (M-mode), time interval from onset of the QRS to aortic valve closure, time interval from onset of the QRS to pulmonary valve opening, right ventricular systolic pressure, LV filling time in relation to cardiac cycle length (pulsed-wave Doppler), E wave, A wave, velocity-time integral (aortic flow), intraventricular delay (tissue Doppler), interventricular delay (tissue Doppler) | ACC/AHA [29,30,33l], Prospect trial [62] |
| Pacemaker Implantation | Procedure performed, time of entry into the operating room*, time of surgical incision/puncture*, time of vascular access obtention*, time of skin closure*, total fluoroscopy time, total ventricular lead positioning time*, total procedure time, pulse generator characteristics, right atrial lead characteristics, right ventricular lead characteristics, left ventricular lead characteristics, crossover*, reason for crossover*, intraoperative complications | ACC/AHA [29], Trials [34-37], *data elements under development |
| Hospital Discharge | Discharge date* (after PM implantation), length of hospital stay (days), medications prescribed, anticoagulation therapy, indication for anticoagulation therapy, postoperative complications, ECG before discharge*, chest radiography before discharge* | ACC/AHA [28-33], Trials [34-37], *data elements under development |
| Follow-up Evaluations | Date of clinical evaluation*, clinical manifestations after PM implantation, functional class (NYHA) after PM implantation, lead-related complications, pulse generator pocket complications (infectious, hemorrhagic, thromboembolic complications), interventions after evaluation*, hospitalization, reason of hospitalization*, treatment during the hospitalization* | ACC/AHA [29], Trials [12,13, 34-37], *data elements under development |
| Pacemaker Interrogation and Programming | Date of PM interrogation, pulse generator battery status, battery longevity, battery voltage, battery impedance, atrial pacing (%), ventricular pacing (%), atrial fibrillation or mode-switch (%), cardiac rhythm, diagnostic counters, right atrial lead threshold/ impedance/ sensing, right ventricular lead threshold/ impedance/ sensing, left ventricular lead threshold/ impedance/ sensing, programmed PM mode, change in the final programming, reason for change in the pacing mode, reason for change in the programmed frequency, minimum heart rate, maximum heart rate, sensor, maximum sensor rate, duration of AV interval after P wave sense, duration of AV interval after atrial pacing, atrial pulse amplitude, ventricular pulse amplitude, conclusion of the lead analysis, conclusion of the device analysis | Data elements under development |
| Heart Failure Biomarkers | Date of biomarkers tests, Natriuretic peptide B, Tumor necrosis factor alpha, Interleukin 6, C reactive protein | Data elements under development |
| Six Minute Walk Distance Test | Date of 6MWD test*, height, weight, BMI, medications taken before the test*, cardiopulmonary parameters before and after 6MWD test, total distance walked in 6 minutes (meters), predicted distance (Iwama and Enright equation), stopped or paused before 6 minutes, reason to stop or pause the test before 6 minutes, symptoms at the end of 6MWD test | ACC/AHA [29], ATS [63], *data elements under development |
| SF-36 Questionnaire | Physical Functioning, Role Physical, Bodily Pain, General Health, Vitality, Social Functioning, Role Emotional, Mental Health, Summary Measures: Physical Health, Summary Measures: Mental Health | ACC/AHA [32], SF-36 [64] |
| Minnesota Living With Heart Failure Questionnaire | Total Score, Emotional domain score, Physical domain score | ACC/AHA [32], MLWHF [65] |
| Aquarel Questionnaire | Chest discomfort domain, Dyspnea and exertion domain, Cognition domain, Arrhythmias domain | Aquarel [66] |
| Completion Data | Date of study completion, follow-up time (enrollment until study completion), anticipated follow-up time (enrollment until study withdraw), reason for patient withdrew from study, lost to follow-up, date of death, autopsy description*, cause of death | CDISC [41], ACC/AHA [28-33], *data elements under development |
| Adverse Events | AE Identifier, date of AE, severity of the AE (persistent or significant disability or incapacity, initial or prolonged hospitalization for the subject, death), relation between AE and study intervention, action take with study treatment, action take in response to this AE, outcome of this AE, date of AE was evaluated by the study committee | ACC/AHA [28-33], CDISC [41] |

AE= adverse event; ACC/AHA= American College of Cardiology/American Heart Association; AV= atrioventricular; ATS= American Thoracic Society; BMI= body mass index; CDISC= Clinical Data Interchange Standards Consortium; ECG= electrocardiogram; INR= International Normalized Ratio; LV= left ventricular; MLWHF= Minnesota Living with Heart Failure questionnaire; NCI= National Cancer Institute; NYHA= New York Heart Association; PM= pacemaker; SF-36= Short-form 36 questionnaire; 6MWD= six minute walk distance

#### 
