# Supplementary material for: Glocal Clinical Registries: Pacemaker Registry Design and Implementation for Global and Local Integration – Methodology and Case Study
Source: PLoS One. 2013 Jul 25;8(7):e71090. doi: 10.1371/journal.pone.0071090 (PMC3723676; doi:10.1371/journal.pone.0071090)
Supplement: Table S2 — Reimbursed values paid by Brazilian government for pacemaker implantation according to Brazilian States. (DOCX) [file pone.0071090.s003.docx]

| **States** | **Reimbursed Values/day (Brazilian real)** | **Reimbursed Values/day (USD)** | **Reimbursed Values/total (Brazilian real)** | **Reimbursed Values/total (USD)** | **Length of hospital stay** |
| --- | --- | --- | --- | --- | --- |
| Distrito Federal | $6,102.00 | $2,856.14 | $8,013.00 | $3,750.61 | 1.96 |
| Goias | $4,826.00 | $2,258.89 | $8,605.00 | $4,027.71 | 3.33 |
| Mato Grosso do Sul | $4,095.00 | $1,916.73 | $8,638.00 | $4,043.15 | 4.08 |
| Mato Grosso | $3,994.00 | $1,869.46 | $8,932.00 | $4,180.77 | 3.59 |
| **Centre west** | **$4,754.25** | **$2,225.30** | **$8,547.00** | **$4,000.56** | **3.24** |
| Alagoas | $3,955.00 | $1,851.20 | $8,435.00 | $3,948.14 | 3.24 |
| Bahia | $5,921.00 | $2,771.42 | $8,237.00 | $3,855.46 | 2.20 |
| Ceara | $3,650.00 | $1,708.44 | $8,035.00 | $3,760.91 | 4.02 |
| Maranhao | $4,705.00 | $2,202.25 | $8,155.00 | $3,817.08 | 3.32 |
| Paraiba | $4,855.00 | $2,272.46 | $8,886.00 | $4,159.23 | 2.78 |
| Pernambuco | $5,355.00 | $2,506.49 | $8,468.00 | $3,963.58 | 3.27 |
| Piaui | $5,669.00 | $2,653.47 | $8,401.00 | $3,932.22 | 1.99 |
| Rio Grande do Norte | $6,304.00 | $2,950.69 | $8,871.00 | $4,152.21 | 2.44 |
| Sergipe | $5,648.00 | $2,643.64 | $8,785.00 | $4,111.96 | 3.51 |
| **Northeast** | **$5,118.00** | **$2,395.56** | **$8,474.78** | **$3,966.76** | **2.97** |
| Acre | $1,824.00 | $853.75 | $8,964.00 | $4,195.74 | 8.25 |
| Amazonas | $3,646.00 | $1,706.57 | $8,497.00 | $3,977.16 | 4.74 |
| Amapa | $1,822.00 | $852.82 | $7,761.00 | $3,632.66 | 5.79 |
| Para | $3,262.00 | $1,526.83 | $8,431.00 | $3,946.26 | 4.51 |
| Rondonia | $931.10 | $435.82 | $4,421.00 | $2,069.32 | 15.85 |
| Tocantins | $3,346.00 | $1,566.15 | $9,174.00 | $4,294.04 | 3.00 |
| **North** | **$2,471.85** | **$1,156.99** | **$7,874.67** | **$3,685.86** | **7.02** |
| Espirito Santo | $4,460.00 | $2,087.57 | $8,448.00 | $3,954.22 | 2.93 |
| Minas Gerais | $4,358.00 | $2,039.83 | $8,626.00 | $4,037.54 | 3.55 |
| Rio de Janeiro | $4,639.00 | $2,171.36 | $8,594.00 | $4,022.56 | 4.57 |
| Sao Paulo | $4,866.00 | $2,277.61 | $8,563.00 | $4,008.05 | 3.83 |
| **Southeast** | **$4,580.75** | **$2,144.09** | **$8,557.75** | **$4,005.59** | **3.72** |
| Parana | $3,813.00 | $1,784.74 | $8,777.00 | $4,108.22 | 4.34 |
| Rio Grande do Sul | $4,720.00 | $2,209.27 | $8,360.00 | $3,913.03 | 3.71 |
| Santa Catarina | $3,891.00 | $1,821.24 | $8,425.00 | $3,943.46 | 4.45 |
| **South** | **$4,141.33** | **$1,938.42** | **$8,520.67** | **$3,988.23** | **4.17** |

*Brazilian real (BRL) converted to US Dollar (USD) in December 2, 2012.

1 BRL = 0.468061 USD

1 USD = 2.13647 BRL
