# Supplementary material for: Glocal Clinical Registries: Pacemaker Registry Design and Implementation for Global and Local Integration – Methodology and Case Study
Source: PLoS One. 2013 Jul 25;8(7):e71090. doi: 10.1371/journal.pone.0071090 (PMC3723676; doi:10.1371/journal.pone.0071090)
Supplement: Table S3 — Cardiac Pacemaker Clinical Trials available at LinkedCT. (DOCX) [file pone.0071090.s004.docx]

| **NCT Number** | **Recruitment** | **Conditions** | **Age Groups** | **Enrollment** | **Sponsor** | **Study Types** |
| --- | --- | --- | --- | --- | --- | --- |
| NCT00215761 | Completed | Atrial Fibrillation | Adult\|Senior | 263 | Other\|Industry | Observational |
| NCT00256152 | Completed | Atrial Fibrillation | Adult\|Senior | 2580 | Industry\|Other | Interventional |
| NCT00262119 | Completed | Atrial Fibrillation | Adult\|Senior | 1166 | Industry | Interventional |
| NCT00419640 | Completed | Atrial Fibrillation | Adult\|Senior | 385 | Industry | Interventional |
| NCT00589303 | Terminated | Atrial Fibrillation | Adult\|Senior | 27 | Other\|Industry | Interventional |
| NCT00187278 | Active, not recruiting | Atrioventricular Block | Adult\|Senior | 1830 | Industry | Interventional |
| NCT00267098 | Active, not recruiting | Atrioventricular Block | Adult\|Senior | 918 | Industry | Interventional |
| NCT00292383 | Recruiting | Atrioventricular Block | Adult\|Senior | 100 | Industry | Interventional |
| NCT00709774 | Not yet recruiting | Atrioventricular Block | Adult\|Senior | 60 | Other | Interventional |
| NCT00925691 | Recruiting | Atrioventricular Block | Adult\|Senior | 180 | Other | Interventional |
| NCT01302717 | Recruiting | Atrioventricular Block | Child\|Adult\|Senior | 98 | Other | Interventional |
| NCT01477658 | Enrolling by invitation | Atrioventricular Block | Child\|Adult\|Senior | 140 | Other | Observational |
| NCT01694550 | Recruiting | Atrioventricular Block | Adult\|Senior | 180 | Other | Observational |
| NCT00000561 | Completed | Bradyarrhythmia | Adult\|Senior |  | NIH | Interventional |
| NCT00116987 | Terminated | Bradyarrhythmia | Senior | 800 | Other | Interventional |
| NCT00133289 | Completed | Bradyarrhythmia | Adult\|Senior | 950 | Industry | Interventional |
| NCT00135174 | Active, not recruiting | Bradyarrhythmia | Adult\|Senior | 1526 | Other | Observational |
| NCT00146861 | Completed | Bradyarrhythmia | Adult\|Senior | 1500 | Industry | Interventional |
| NCT00156741 | Active, not recruiting | Bradyarrhythmia | Adult\|Senior | 150 | Industry | Interventional |
| NCT00170326 | Completed | Bradyarrhythmia | Adult\|Senior | 108 | Industry | Interventional |
| NCT00275769 | Completed | Bradyarrhythmia | Child\|Adult\|Senior | 860 | Industry | Interventional |
| NCT00292539 | Completed | Bradyarrhythmia | Child\|Adult\|Senior | 600 | Industry | Observational |
| NCT00294034 | Completed | Bradyarrhythmia | Child\|Adult\|Senior | 950 | Industry | Observational |
| NCT00307073 | Completed | Bradyarrhythmia | Child\|Adult\|Senior | 120 | Industry | Interventional |
| NCT00311168 | Completed | Bradyarrhythmia | Adult\|Senior | 100 | Industry | Interventional |
| NCT00382525 | Completed | Bradyarrhythmia | Child\|Adult\|Senior | 8586 | Industry | Observational |
| NCT00704093 | Completed | Bradyarrhythmia | Adult\|Senior | 10 | Other | Interventional |
| NCT00976482 | Active, not recruiting | Bradyarrhythmia | Adult\|Senior | 1730 | Industry | Observational |
| NCT00989326 | Completed | Bradyarrhythmia | Adult\|Senior | 543 | Industry | Interventional |
| NCT01000532 | Completed | Bradyarrhythmia | Adult\|Senior | 88 | Industry | Observational |
| NCT01030705 | Completed | Bradyarrhythmia | Adult\|Senior | 80 | Industry\|Other | Observational |
| NCT01038180 | Completed | Bradyarrhythmia | Child\|Adult\|Senior | 28000 | Other | Observational |
| NCT01044407 | Recruiting | Bradyarrhythmia | Adult\|Senior | 14 | Other | Interventional |
| NCT01062126 | Completed | Bradyarrhythmia | Adult\|Senior | 3389 | Industry | Observational |
| NCT01076374 | Recruiting | Bradyarrhythmia | Child\|Adult\|Senior | 336 | Industry | Observational |
| NCT01095770 | Not yet recruiting | Bradyarrhythmia | Child\|Adult\|Senior | 50 | Other | Interventional |
| NCT01163422 | Enrolling by invitation | Bradyarrhythmia | Adult\|Senior | 20 | Other\|Industry | Interventional |
| NCT01219621 | Completed | Bradyarrhythmia | Adult\|Senior | 450 | Industry | Interventional |
| NCT01522755 | Recruiting | Bradyarrhythmia | Adult\|Senior | 700 | Industry | Observational |
| NCT01535404 | Recruiting | Bradyarrhythmia | Adult\|Senior | 190 | Other\|Industry | Interventional |
| NCT01559311 | Recruiting | Bradyarrhythmia | Adult\|Senior | 177 | Industry | Interventional |
| NCT01647490 | Recruiting | Bradyarrhythmia | Adult\|Senior | 408 | Other | Interventional |
| NCT01688843 | Recruiting | Bradyarrhythmia | Adult\|Senior | 1030 | Industry | Interventional |
| NCT01700244 | Recruiting | Bradyarrhythmia | Adult\|Senior | 36 | Industry | Interventional |
| NCT01715558 | Not yet recruiting | Bradyarrhythmia | Adult\|Senior | 500 | Industry | Observational |
| NCT01717469 | Recruiting | Bradyarrhythmia | Adult\|Senior | 12 | Other | Interventional |
| NCT01803217 | Active, not recruiting | Bradyarrhythmia | Adult\|Senior | 70 | Other\|Industry | Interventional |
| NCT01819662 | Not yet recruiting | Bradyarrhythmia | Child\|Adult\|Senior | 2100 | Other | Interventional |
| NCT00161551 | Recruiting | Bradyarrhythmia/Sick Sinus Syndrome | Adult\|Senior | 402 | Industry | Interventional |
| NCT00224341 | Recruiting | Bradyarrhythmia/Sick Sinus Syndrome | Adult\|Senior | 360 | Industry | Interventional |
| NCT00228241 | Active, not recruiting | Bradyarrhythmia/Sick Sinus Syndrome | Adult\|Senior | 100 | Other | Interventional |
| NCT00236158 | Terminated | Bradyarrhythmia/Sick Sinus Syndrome | Adult\|Senior | 1415 | Other | Interventional |
| NCT00239226 | Not yet recruiting | Bradyarrhythmia/Sick Sinus Syndrome | Adult\|Senior | 300 | Industry | Interventional |
| NCT00284830 | Completed | Bradyarrhythmia/Sick Sinus Syndrome | Adult\|Senior | 1070 | Industry | Interventional |
| NCT00475124 | Active, not recruiting | Bradyarrhythmia/Sick Sinus Syndrome | Adult\|Senior | 123 | Industry | Interventional |
| NCT00531037 | Active, not recruiting | Bradyarrhythmia/Sick Sinus Syndrome | Child\|Adult\|Senior | 1440 | Industry | Observational |
| NCT01074749 | Recruiting | Bradyarrhythmia/Sick Sinus Syndrome | Adult\|Senior | 158 | Industry | Interventional |
| NCT01170611 | Completed | Bradyarrhythmia/Sick Sinus Syndrome | Adult\|Senior | 400 | Industry | Interventional |
| NCT01294839 | Recruiting | Bradyarrhythmia/Sick Sinus Syndrome | Adult\|Senior | 555 | Industry | Interventional |
| NCT01441583 | Active, not recruiting | Bradyarrhythmia/Sick Sinus Syndrome | Adult\|Senior | 139 | Industry | Interventional |
| NCT01477138 | Recruiting | Bradyarrhythmia/Sick Sinus Syndrome | Adult\|Senior | 126 | Other | Observational |
| NCT01611389 | Recruiting | Bradyarrhythmia/Sick Sinus Syndrome | Adult\|Senior | 60 | Other | Interventional |
| NCT00156390 | Completed | Heart Failure | Adult\|Senior | 187 | Other | Interventional |
| NCT00157846 | Terminated | Heart Failure | Adult\|Senior | 100 | Industry | Interventional |
| NCT00158938 | Completed | Heart Failure | Adult\|Senior | 115 | Industry | Interventional |
| NCT00158964 | Completed | Heart Failure | Adult\|Senior | 110 | Industry | Interventional |
| NCT00170300 | Completed | Heart Failure | Adult\|Senior | 813 | Industry | Interventional |
| NCT00180258 | Completed | Heart Failure | Adult\|Senior | 2200 | Industry | Interventional |
| NCT00187213 | Completed | Heart Failure | Adult\|Senior |  | Industry | Interventional |
| NCT00187252 | Completed | Heart Failure | Adult\|Senior | 380 | Industry | Interventional |
| NCT00199498 | Active, not recruiting | Heart Failure | Adult\|Senior | 160 | Other | Interventional |
| NCT00205192 | Completed | Heart Failure | Child\|Adult\|Senior | 100 | Other | Observational |
| NCT00221780 | Recruiting | Heart Failure | Adult\|Senior | 33 | Other | Interventional |
| NCT00253357 | Completed | Heart Failure | Adult\|Senior | 450 | Industry | Observational |
| NCT00269230 | Completed | Heart Failure | Adult\|Senior | 441 | Industry | Interventional |
| NCT00271544 | Completed | Heart Failure | Adult\|Senior | 190 | Industry | Interventional |
| NCT00321295 | Completed | Heart Failure | Adult\|Senior | 40 | Other | Interventional |
| NCT00370526 | Terminated | Heart Failure | Adult\|Senior | 100 | Industry | Interventional |
| NCT00376116 | Completed | Heart Failure | Adult\|Senior | 513 | Industry | Observational |
| NCT00384722 | Completed | Heart Failure | Adult\|Senior | 70 | Industry | Interventional |
| NCT00385749 | Recruiting | Heart Failure | Adult\|Senior | 98 | Other\|Industry | Interventional |
| NCT00389649 | Completed | Heart Failure | Adult\|Senior | 24 | Other | Interventional |
| NCT00399594 | Active, not recruiting | Heart Failure | Adult\|Senior | 300 | Other\|Industry | Interventional |
| NCT00420108 | Completed | Heart Failure | Adult\|Senior | 23 | Other\|Industry | Observational |
| NCT00461734 | Active, not recruiting | Heart Failure | Adult\|Senior | 248 | Industry | Interventional |
| NCT00480051 | Recruiting | Heart Failure | Adult\|Senior | 60 | Other | Interventional |
| NCT00551681 | Recruiting | Heart Failure | Adult\|Senior | 52 | Other | Interventional |
| NCT00577187 | Withdrawn | Heart Failure | Child\|Adult\|Senior | 0 | Industry | Interventional |
| NCT00670111 | Terminated | Heart Failure | Adult\|Senior | 400 | Industry | Interventional |
| NCT00748735 | Completed | Heart Failure | Adult\|Senior | 55 | Other | Observational |
| NCT00812201 | Completed | Heart Failure | Adult\|Senior | 80 | Industry | Observational |
| NCT00814840 | Active, not recruiting | Heart Failure | Adult\|Senior | 100 | Other | Interventional |
| NCT00825006 | Recruiting | Heart Failure | Adult\|Senior | 30 | Other | Interventional |
| NCT00833352 | Active, not recruiting | Heart Failure | Adult\|Senior | 263 | Industry | Interventional |
| NCT00853593 | Completed | Heart Failure | Adult\|Senior | 197 | Industry | Interventional |
| NCT00875732 | Recruiting | Heart Failure | Adult\|Senior | 40 | Other\|Industry | Interventional |
| NCT00905944 | Recruiting | Heart Failure | Adult\|Senior | 45 | Other | Interventional |
| NCT00927251 | Completed | Heart Failure | Child\|Adult\|Senior | 90 | Industry | Interventional |
| NCT00941850 | Recruiting | Heart Failure | Adult\|Senior | 88 | Other | Interventional |
| NCT00944125 | Active, not recruiting | Heart Failure | Adult\|Senior | 50 | Other\|Industry | Interventional |
| NCT01008670 | Completed | Heart Failure | Adult\|Senior | 120 | Industry | Observational |
| NCT01016431 | Recruiting | Heart Failure | Adult\|Senior | 20 | Other | Interventional |
| NCT01035489 | Active, not recruiting | Heart Failure | Adult\|Senior | 85 | Other | Interventional |
| NCT01055210 | Not yet recruiting | Heart Failure | Adult\|Senior | 40 | Other | Interventional |
| NCT01059175 | Active, not recruiting | Heart Failure | Adult\|Senior | 84 | Industry\|Other | Interventional |
| NCT01193712 | Recruiting | Heart Failure | Adult\|Senior | 40 | Other | Interventional |
| NCT01241838 | Terminated | Heart Failure | Adult\|Senior | 11 | Other | Interventional |
| NCT01242722 | Completed | Heart Failure | Adult\|Senior | 100 | Industry | Observational |
| NCT01260402 | Recruiting | Heart Failure | Adult\|Senior | 80 | Other | Interventional |
| NCT01270646 | Recruiting | Heart Failure | Adult\|Senior | 40 | Other | Interventional |
| NCT01277783 | Enrolling by invitation | Heart Failure | Adult\|Senior | 130 | Industry | Interventional |
| NCT01294527 | Active, not recruiting | Heart Failure | Adult\|Senior | 100 | Industry | Interventional |
| NCT01296282 | Not yet recruiting | Heart Failure | Child\|Adult\|Senior | 50 | Other | Observational |
| NCT01302470 | Completed | Heart Failure | Adult\|Senior | 30 | Other | Interventional |
| NCT01365234 | Active, not recruiting | Heart Failure | Adult\|Senior | 40 | Industry | Interventional |
| NCT01417624 | Recruiting | Heart Failure | Adult\|Senior | 70 | Other | Interventional |
| NCT01420393 | Recruiting | Heart Failure | Adult\|Senior | 1000 | Other | Interventional |
| NCT01446796 | Terminated | Heart Failure | Adult\|Senior | 3 | Other\|Industry | Interventional |
| NCT01484613 | Recruiting | Heart Failure | Adult\|Senior | 337 | Industry | Observational |
| NCT01512381 | Recruiting | Heart Failure | Adult\|Senior | 60 | Other | Interventional |
| NCT01539629 | Completed | Heart Failure | Adult\|Senior | 50 | Industry | Observational |
| NCT01586884 | Recruiting | Heart Failure | Adult\|Senior | 10 | Other | Interventional |
| NCT01609738 | Recruiting | Heart Failure | Adult\|Senior | 12 | Other\|Industry | Observational |
| NCT01652248 | Completed | Heart Failure | Adult\|Senior | 50 | Other | Interventional |
| NCT01660035 | Recruiting | Heart Failure | Child\|Adult\|Senior | 500 | Industry | Observational |
| NCT01735916 | Recruiting | Heart Failure | Adult\|Senior | 2900 | Industry | Interventional |
| NCT01751022 | Recruiting | Heart Failure | Adult\|Senior | 1210 | Industry | Interventional |
| NCT01798043 | Recruiting | Heart Failure | Adult\|Senior | 50 | Industry | Observational |
| NCT01818765 | Not yet recruiting | Heart Failure | Adult\|Senior | 20 | Other | Interventional |
| NCT01831518 | Not yet recruiting | Heart Failure | Adult\|Senior | 30 | Other\|Industry | Interventional |
